# Transcriptomic Analyses Unveil Hydrocarbon Degradation Mechanisms in a Novel Polar *Rhodococcus* sp. Strain R1B_2T From a High Arctic Intertidal Zone Exposed to Ultra‐Low Sulphur Fuel Oil

**DOI:** 10.1111/1758-2229.70218

**Published:** 2025-10-16

**Authors:** Nastasia J. Freyria, Antoine‐Olivier Lirette, Brady R. W. O'Connor, Charles W. Greer, Lyle G. Whyte

**Affiliations:** ^1^ Department of Natural Resource Sciences, Faculty of Agricultural and Environmental Sciences McGill University Quebec Canada; ^2^ Graduate School of Agriculture Hokkaido University Sapporo Japan; ^3^ Energy, Mining and Environment Research Centre National Research Council of Canada Montreal Quebec Canada

**Keywords:** Arctic bacteria, biodegradation, comparative transcriptomic, differential gene expression, hydrocarbon degradation, *Rhodococcus*

## Abstract

As Arctic shipping increases due to climate change, characterised by rising temperatures and decreasing sea‐ice coverage, the risk of oil spills through the Northwest Passage in this fragile ecosystem grows, necessitating effective bioremediation strategies. Research on bioremediation using Arctic coastal sediment bacteria has gained attention, particularly *Rhodococcus* species that play key roles in hydrocarbon degradation under extreme conditions. This study investigates the hydrocarbon degradation capabilities of a novel cryophilic Arctic *Rhodococcus* sp. strain R1B_2T isolated from Canadian high Arctic beach sediment in Resolute Bay, exposed to ultra‐low sulphur fuel oil for 3 months at 5°C. Comparative transcriptomics analyses revealed dynamic responses and metabolic plasticity, with upregulation of genes for aliphatic, aromatic, and polycyclic aromatic hydrocarbons, biosurfactant production (e.g., rhamnolipid), cold adaptation, and stress responses. The strain possesses several key alkane degradation genes (*alkB, almA, CYP153, ladA*), with co‐expression network analysis highlighting synergistic mechanisms between *alkB* and *CYP153* that target different chain‐length alkanes (*alkB*: ~C5–C20; *CYP153*: ~C5–C12 and > C30), demonstrating complementary degradation strategies. The findings reveal adaptive mechanisms and degradation kinetics of native Arctic bacteria, highlighting the potential of Arctic cryophilic and halotolerant *Rhodococcus* species for oil spill remediation in polar marine environments.

## Graphical abstract

Cold‐adapted *Rhodococcus* sp. strain R1B_2T from Arctic Tupirvik Beach (Northwest Passage) degrades short‐ to long‐chain hydrocarbons in summer seawater (−1°C to 5°C) via a synergistic activity of key hydrocarbon degradation genes (*alkB*, *CYP153*, *almA*, and *ladA*), with rhlABCR‐linked rhamnolipid production supporting biodegradation.

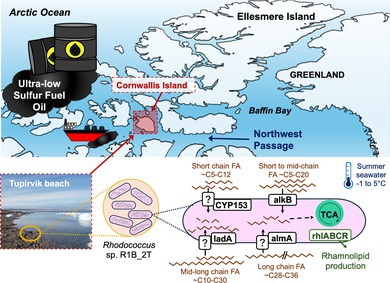

## 1 Introduction

The rapidly warming Arctic is thinning and reducing sea ice, opening the Northwest Passage (NWP) to increased shipping (Dawson et al. 2018). With this accessibility comes a heightened risk of oil pollution, especially from heavy fuel oil (HFO), in a pristine and fragile ecosystem (Elbaz et al. 2015; Nevalainen et al. 2017; Reddy et al. 2018). Despite emerging International Maritime Organisation restrictions on HFO use (Comer et al. 2020), effective and cold‐adapted remediation remains urgently needed. Indigenous communities have called for stronger protection of their waters and lands (Prior and Walsh 2018; Dawson et al. 2020), underscoring both governance gaps and practical response needs. Given the increasing risk of oil spills in the Arctic and the limitations of conventional remediation approaches in this extreme and remote environment, research into the hydrocarbon degradation capabilities of Arctic coastal sediment bacteria has become paramount for developing effective bioremediation strategies. Arctic coastal sediments harbour diverse microbial communities adapted to extreme cold and fluctuating environmental conditions (Müller et al. 2018), potentially offering native biological solutions for oil contamination that could complement or enhance traditional cleanup methods. These indigenous microorganisms may possess unique metabolic capabilities that enable effective hydrocarbon degradation under conditions where mechanical recovery or chemical dispersants face important logistical challenges.

Cold‐adapted hydrocarbon degraders with demonstrated efficiency include a variety of well‐characterised strains. The psychrotrophic strains BI7 and BI8 of *Pseudomonas* sp. possess the *alkB* and *nah* pathways, which are responsible for the biodegradation of alkanes and naphthalene at 5°C (Whyte et al. 1997). *Oleispira antarctica* RB‐8 rapidly removes n‐alkanes at 4°C–10°C and often dominates low‐temperature alkane enrichments (Gregson et al. 2020). Psychrotolerant *Rhodococcus* species, such as *R. qingshengii* TUHH‐12, degrade medium‐long chain alkanes at 4°C–15°C (Lincoln et al. 2015). *Cycloclasticus* sp. 78‐ME oxidises polycyclic aromatic hydrocarbons (PAHs) at 4°C–8°C (Messina et al. 2016), while *Glaciecola psychrophila* 170T produces cold‐active aromatic‐catabolic enzymes (Zhang et al. 2006). In addition, *Marinobacter* sp. BSs20148 degrades C10‐C20 alkanes from Arctic seawater (Song et al. 2013), and *Polaromonas naphthalenivorans* CJ2 mineralizes naphthalene at ≤ 10°C (Jeon et al. 2006; Jin et al. 2016). Despite this demonstrated low‐temperature performance, most studies still test single substrates, leaving the regulation of pathways in complex fuel mixtures such as ultra‐low sulphur fuel oil (ULSFO) underexplored. High‐throughput ‐omics now clarify the mechanisms underlying cold‐environment oil degradation. Metagenomics, transcriptomics, and metabolomics reveal upregulation of alkane monooxygenases (*alkB*/*almA*), β‐oxidation, PAH ring‐dioxygenases, and catechol‐cleavage pathways, alongside genes for biosurfactants and cold adaptation, in isolates and communities exposed to oil (Kimes et al. 2013; Qin et al. 2014; Cowan et al. 2015; Laczi et al. 2015; Messina et al. 2016; Conte et al. 2018; Gregson et al. 2020; Freyria, Góngora, et al. 2024; Lirette et al. 2024). However, the regulation of these pathways in response to complex fuel mixtures remains unclear in polar systems.

Long‐term studies in Arctic environments face extreme weather, logistical constraints, and environmental sensitivities (Vergeynst et al. 2018), yet they are crucial for understanding and protecting these fragile ecosystems (Vincent et al. 2011). The Baffin Island Oil Spill (BIOS) study (Owens et al. 1987; Sergy and Blackall 1987; Hunnie et al. 2023; Schreiber et al. 2023) exemplifies this value, providing four decades of insight into the fate and effects of oil in Arctic marine systems. Combining in situ observations with laboratory experiments and high‐throughput analyses can yield a comprehensive view of Arctic microbial oil degradation, informing predictive models and response strategies where conventional clean‐up methods are often constrained (Vergeynst et al. 2018).

Here, we investigate hydrocarbon degradation by a novel Arctic *Rhodococcus* strain R1B_2T, a psychrotolerant bacterium (0°C–25°C), isolated from high Arctic NWP beach sediment, Resolute Bay, Nunavut, Canada (Lirette et al. 2024). In recent metagenomic and culture‐based surveys of NWP marine beach sediments (Chen et al. 2024; Freyria, Góngora, et al. 2024; Góngora et al. 2024; Lirette et al. 2024), *Rhodococcus* emerges as a predominant hydrocarbon degrader (Whyte et al. 1998; Whyte et al. 2002) and a dominant genus in oil‐contaminated Arctic beach sediments (Whyte et al. 2002), highlighting its potential for in situ bioremediation (Whyte et al. 2001). Here, we assess the capacity of strain R1B_2T to degrade aliphatic, aromatic, and PAHs and identify the key genes and metabolic pathways involved. We exposed R1B_2T to ULSFO as the sole carbon source for three months at 5°C in liquid culture and conducted comparative transcriptomics to reveal its pathway‐level responses and metabolic plasticity in cold marine conditions.

## 2 Experimental Procedures

### 2.1 Strain Isolation and Growth Conditions

Lirette (2023) isolated *Rhodococcus* sp. strain R1B_2T. Briefly, sediments (5 g) from a 1‐month column experiment simulating the intertidal cycle in Arctic beach sediment (Chen et al. 2024) were placed in 50 mL Falcon tubes, which were then supplemented with sterilised glass beads and water (3 times the sediment weight, 1:4 dilution). Each tube was vortexed for 2 min to dislodge the sediments. Subsequently, 1 mL of each solution was added to 9 mL of autoclave distilled water, achieving a 10‐fold dilution. This process continued until a 10^−5^ dilution was reached. Then, 100 *μ*L of supernatant from the 10^−3^, 10^−4^ and 10^−5^ dilutions were spread on two types of plates: Reasoner's 2A (R2A, BD) gellan plates (Alfa Aesar), which support slow‐growing microorganisms (Reasoner and Geldreich 1985), and plates made of autoclaved beach water (from Tupirvik beach, Resolute Bay), 2% gellan gum (Alfa Aesar), and 0.67 g·L^−1^ ammonium sulphate amended with 500 ppm ULSFO (Lirette 2023).

To determine hydrocarbon biodegradation, ULSFO was added to a final concentration of 500 ppm (0.045 g of ULSFO per 100 mL) of sterile artificial seawater (3% sea salt), with triplicates for 3 conditions: (i) negative control culture without *Rhodococcus* cells and supplemented with 500 ppm of ULSFO and 0.35 g·L^−1^ ammonium sulphate; (ii) control culture of *Rhodococcus* cells without ULSFO and supplemented with R2A broth and 0.35 g·L^−1^ ammonium sulphate; (iii) and (iv) culture of *Rhodococcus* cells supplemented with 500 ppm of ULSFO and 0.35 g·L^−1^ ammonium sulphate. Cultures of *Rhodococcus* under all conditions were incubated for 1 and 3 months on shakers at 100 rpm in a 5°C incubator (Lirette 2023). The initial concentration of cells was 10^4^ cells·mL^−1^. Cells were collected after each time point (T0 = initial time point, T1 = after 1 month, and T3 = after 3 months) in 50 mL falcon tubes, centrifuged for 2 min at 5000 rpm, and supernatant was removed each time. Pellets were resuspended in 2 mL of Zymo Research DNA/RNA Shield (Irvine, CA, USA), incubated for 30 min at room temperature and stored at −80°C until processed.

### 2.2 Petroleum Hydrocarbon Analyses

After each incubation period, the medium was heated to 60°C to suspend ULSFO adhering to the glass flask, then transferred to glass bottles provided by SGS Canada (Montreal, Quebec) and stored at −20°C until all samples were collected for petroleum hydrocarbon analysis. For aqueous samples, the extraction of semi‐volatile organic compounds (SVOCs) was carried out using dichloromethane by liquid–liquid extraction to ensure complete removal of the analytes from the water, and the quantification of SVOCs was conducted using the United States Environmental Protection Agency (EPA) Method 8270E (EPA 2014). Petroleum hydrocarbons, including PAHs and aliphatic hydrocarbons, were analysed according to SGS Canada's protocols and the CCME Reference Method for Petroleum Hydrocarbons in Soil—Tier 1 (Canadian Council of Ministers of the Environment (CCME) 2002). The initial concentration of each hydrocarbon group in ULSFO was determined by employing negative controls of culture without cells as they demonstrated no indications of degradation. The hydrocarbon removal ratio was determined by subtracting the measured amounts of each group in the remaining oil and liquid culture from the initial concentration in ULSFO (Table S1). This result was divided by the concentration of that group in ULSFO and multiplied by 100 to obtain the percentage of hydrocarbon removal (Chen et al. 2024). All experimental samples were analysed with biological triplicates (three independent cultures), each with technical duplicates (*n* = 6 total measurements per condition). The negative control (medium with oil but no cells, T0) was analysed with technical duplicates only. Error bars represent the standard deviation across all replicates for each condition.

### 2.3 DNA Extraction, Whole‐Genome Library Preparation, Sequencing, and Processing

Whole genome extraction was conducted using the DNeasy PowerSoil DNA extraction kit (Qiagen) according to the manufacturer's instructions. Whole genome sequencing was performed with DNA shotgun (with PCR) following the sample preparation guidelines: 50 μL of solution containing 150 ng of nucleic acid was added to a 96‐well plate. Library preparation was performed using the NEBNext Ultra II DNA Library Prep Kit for Illumina according to the manufacturer's protocol. DNA concentrations were verified with a Qubit fluorometer (Life Technologies, Invitrogen) using the dsDNA Kit (Invitrogen) and with a NanoDrop 8000 spectrophotometer (Thermo Scientific). The sequencing was performed at Genome Quebec (Montréal, Canada) using the Illumina NovaSeq 6000 S4 PE150 platform, resulting in the generation of 35M reads. Raw reads were trimmed using Trimmomatic v0.36 (Bolger et al. 2014) and assembled with Spades v3.15.4 (Bankevich et al. 2012). Assembled reads were annotated using METAerg (Dong and Strous 2019) for Kyoto Encyclopedia of Genes and Genomes (KEGG) pathways (Kanehisa and Goto 2000). The Calgary approach to annotating hydrocarbon (CANT‐HYD) (Khot et al. 2022) was used to detect hydrocarbon degradation genes with an E‐value cut‐off of 0.01. Assembled reads were also submitted to JGI for inclusion in the integrated microbial genomes (IMG) database and functional annotation (Markowitz et al. 2007).

### 2.4 RNA Extraction, Library Preparation, Sequencing, and Processing

RNA from cells was extracted using the RNeasy mini kit (Qiagen) according to the manufacturer's instructions. Quantification of RNA was verified using a Qubit fluorometer with the RNA BR Assay Kit, and the quality was checked with a NanoDrop 8000 spectrophotometer. Sequencing libraries were prepared with the NEBNext rRNA Depletion kit (Bacteria, New England BioLabs). The DNA libraries were then pooled equimolarly for normalisation. The quality and quantity of the pooled libraries were verified using the Agilent High Sensitivity DNA kit on a 2100 Bioanalyzer (Agilent Technologies, Santa Clara, CA, USA). The indexed and pooled final library was sequenced on an Illumina NexSeq Illumina sequencer with a PE100 flow cell at Genome Quebec Innovation Center, resulting in the generation of 400M reads.

### 2.5 Transcriptome Data Processing and Experimental Design

The experiment involved 15 samples, derived from three different time points (T0, T1, and T3), each with three replicates. These samples were split across three conditions: the control group with cells and without ULSFO at T0, T1, and T3, and cells exposed to ULSFO at T1 and T3. The following analyses were conducted using Compute Canada (Digital Research Alliance) facilities and in‐house computers. Raw sequences were quality checked at Genome Quebec and were trimmed in‐house using BBMap v.38.18 (Bushnell 2014) with paired‐end mode. All trimmed transcriptome reads were mapped separately onto the *Rhodococcus* sp. strain R1B_2T reference genome (Lirette et al. 2024) available at the JGI (Joint Genome Institute) Genome Portal using Spliced Transcripts Alignment to a Reference (STAR) v.2.7.10b (Dobin and Gingeras 2015) with default parameters. The mapped reads were counted to evaluate the number of aligned read pairs to each gene between biological replicates. Overall, transcriptome reads mapped to 4395 genes in the *Rhodococcus* sp. strain R1B_2T reference genome. The total number of clean reads generated for all samples was 697,108. After the number of reads per gene was determined for each transcriptome, sequencing replicates were pooled (Table S2).

Gene annotations were retrieved from the JGI Genome Portal with *Rhodococcus* sp. strain R1B_2T as the reference genome. Gene annotations included those from Gene Ontology (GO), KEGG, eukaryotic Ortholog Groups of proteins (KO), Clusters of Orthologous Genes (COG), signal peptide and Interproscan annotations. Carbohydrate‐active enzymes (CAZyme) were predicted using dbCAN, a web server for automated CAZyme annotation using hmmscan v.3.3.2 and based on the CAZy database (Cantarel et al. 2009). The CANT‐HYD (Khot et al. 2022) was utilised to detect the presence of marker genes among all transcriptome reads, with a ‘noise’ cut‐off score. Hydrocarbon aerobic degradation enzymes and genes (HADEG) (Rojas‐Vargas et al. 2023) was also used to detect marker genes. Both CANT‐HYD and HADEG enable the identification of genes involved in both aerobic and anaerobic hydrocarbon degradation pathways, encompassing aliphatic and aromatic hydrocarbons.

### 2.6 Phylogeny and Pangenome Comparison

A phylogenetic tree was constructed as previously described (Freyria 2021; Freyria et al. 2021) from curated full‐length 16S rRNA gene sequences of the several known species of *Rhodococcus* using randomised accelerated maximum likelihood (RAxML) v.8.2.11, 1000 bootstrap replicates, and using the model GTRGAMMA (Stamatakis 2014). Sequences were aligned using MUSCLE (v.3.8.31) (Edgar 2004). The genome of *R*. sp. strain R1B_2T (Lirette 2023) was compared to the genome of *R. cercidiphylli* strain IEGM 1322 (RefSeq: GCF_033042225.1) using OrthoANI calculations from EzBioCloud (Chalita et al. 2024). The average nucleotide identity (ANI) analysis was performed using fastANI v.1.34 (Hernández‐Salmerón and Moreno‐Hagelsieb 2022) by comparing 8 genomes of *Rhodococcus* species and the genome of *Rhodococcus* sp. strain R1B_2T (Table S3).

Pangenome analysis was performed using Roary v.3.13.0 (Page et al. 2015) by comparing the 9 mentioned genomes, following rapid functional annotation using Prokka v.1.14.6 (Seemann 2014). To examine the evolutionary relationships of key genes involved in hydrocarbon degradation, we constructed phylogenetic trees for *alkB* (using MUSCLE alignment and RAxML with the GTRGAMMA model) and *almA* genes. For *almA*, putative homologues were identified by blastp against the known *almA* gene from *Alloalcanivorax dieselolei* (ADP30851.1; Table S4), and a phylogenetic tree was constructed from the highest‐scoring sequences using RAxML with the model PROGAMMALG and 1000 bootstrap replicates.

### 2.7 Statistical Analyses

All further analyses were performed in the RStudio v.4.3.3 environment. Counted mapped reads of all transcriptomes were normalised using *DESeq2* package in R (Love et al. 2014), where counts are divided by sample‐specific size factors determined by the median ratio of gene counts. This step was followed by differential gene expression analyses using *DESeq2*. Multiple hypothetical testing was corrected by the Benjamini‐Hochberg method (Benjamini and Hochberg 2018) in the *DESeq2* package. Genes with Benjamini‐Hochberg‐adjusted *p* value ≤ 0.05 and an absolute fold change (FC) ≥ 2 (i.e., |log2FC| ≥ 1) were considered as significant differentially expressed genes (DEGs). These DEGs were observed between the different sampling times (T0, T1, and T3) and between the control (without oil) and the oil incubation conditions. The representation of DEGs between samples was visualised using scatter plots as previously described (Freyria et al. 2022).

A two‐way analysis of variance (ANOVA) using Past4 v.4.11 software, but with repeated measure was conducted to analyse the time effect between conditions on TPH analysis. Canonical Correspondence Analysis was computed using the *cca()* function from the *vegan* package (Dixon 2003) to discriminate different transcriptomes for each condition according to the time of sampling and the effect of the presence of ULSFO. A dendrogram was computed using the *hclust()* function from the *Stats* package (R Core Team 2018). The complete linkage method was used to construct the dendrogram, which finds similar clusters among samples. The top 25 DEG heatmap was computed on normalised and transformed read counts with the *vst()* function in *Deseq2* and visualised with the *pheatmap()* function from the *pheatmap* package (Kolde and Kolde 2015). Circular heatmaps of selected DEGs were based on the normalised number of reads per gene and on values of log_2_ FC between comparison of time of sampling among all conditions, and were visualised using the *circos.heatmap()* function in R from the *circlize* package (Gu et al. 2014). To visualise the DEGs with differential gene expression relative to the log‐fold change, we used scatter plots and volcano plots computed with the *DESeq2* package.

We performed weighted gene co‐expression network analysis (WGCNA) using the *WGCNA* package (Langfelder and Horvath 2008) in R on DEGs between conditions to analyse conditions of incubation of ULSFO and time effects as previously described (Freyria, de Oliveira, et al. 2024). To construct co‐expression modules, we used default settings except with a soft thresholding power of 15, a minimum module size of 50 genes, and a branch merge cut height of 0.25. Genes were clustered into 4 correlated modules. To identify the relationship between the time of sampling and oil conditions within all modules, Pearson's correlation coefficient was calculated. Hub genes with the highest connectivity among each module were calculated using intramodular connectivity (K.in) and module correlation degree (MM) of each gene. Hub genes may represent key genes with potentially important functions. Module membership (MM) versus gene significance (GS) was statistically investigated in four modules (brown, blue, turquoise and grey) via the WGCNA package. MM refers to the strength of the association between a gene and a module. GS is defined as the correlation between gene expression and stress status. Networks of each module were visualised using Cytoscape v.3.9.1 (Shannon et al. 2003). The node circle size is positively correlated with the number of genes that are partnered within interactions.

## 3 Results

### 3.1 Taxonomic Assignment and Genomic Context of *Rhodococcus* sp. Strain R1B_2T

**FIGURE 1:**
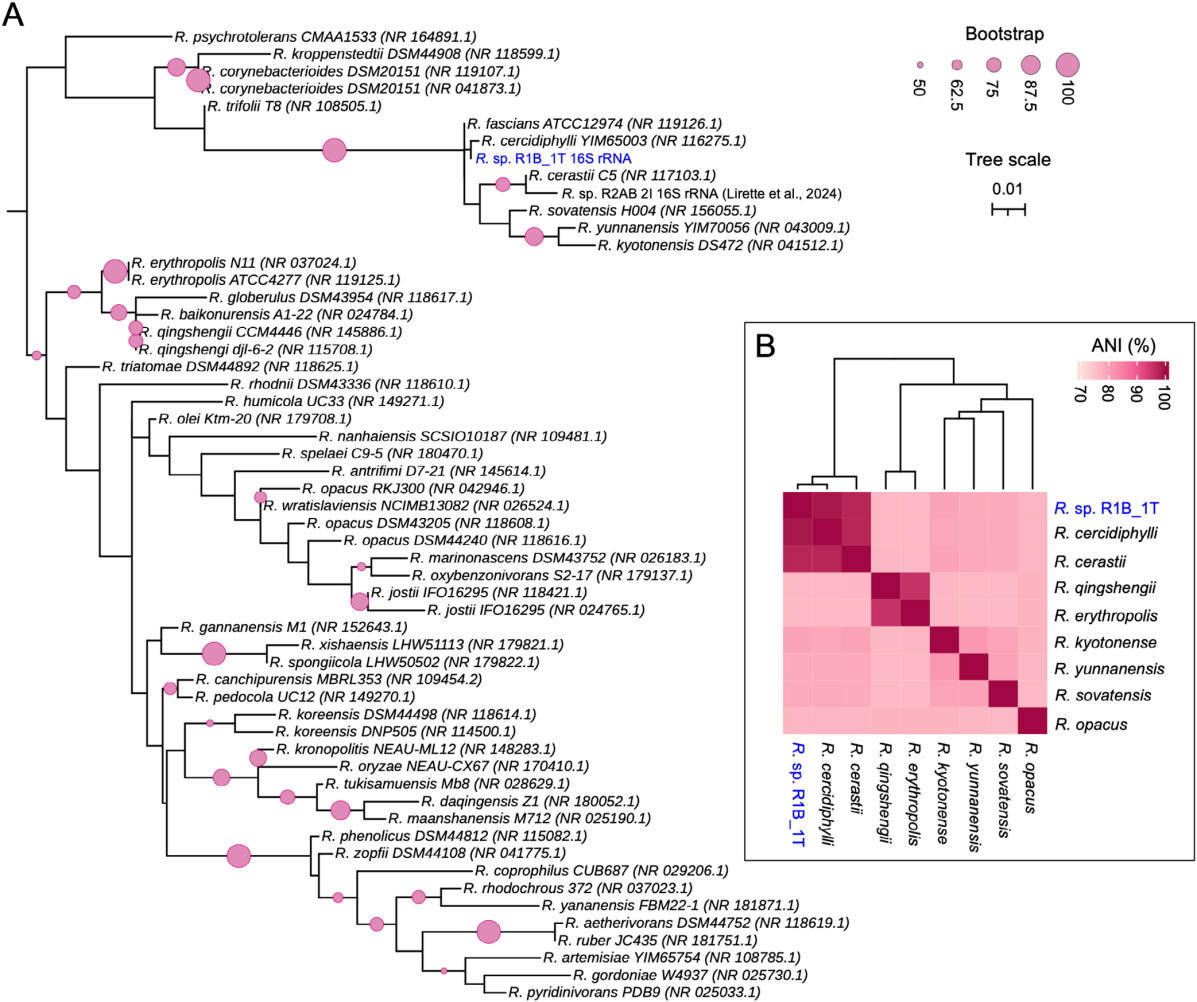
Novel strain R1B_2T phylogenetic position compared to 55 other species of *Rhodococcus*. (A) A Maximum Likelihood tree generated using RAxML with 16S rRNA gene sequences of 55 strains and species of *Rhodococcus* was aligned along with one reference sequence of a short length 16S rRNA gene (sequenced using the Sanger method). The phylogenetic tree was constructed from an alignment of 56 sequences, spanning 909 bp, with bootstrap support calculated over 1000 repetitions. Only bootstrap values greater than 50 (out of 100) are displayed. (B) Heatmap of average nucleotide identity (ANI) between 8 referenced genomes of *Rhodococcus* species and novel strain R1B_2T. ANI and genome information can be found in Table S3.

We assessed the taxonomic position of *Rhodococcus* sp. strain R1B_2T using phylogenetic and genomic analyses. A 16S rRNA maximum‐likelihood tree (RAxML) placed R1B_2T within the *R*. *cercidiphylli–R. cerastii* clade (bootstrap = 85 for the broader cluster), while fine‐scale branching between R1B_2T and *R. cercidiphylli* was unresolved; the isolate was clearly distinct from polar species *R. erythropolis* and *R. qingshengii* (Figure 1A). Whole‐genome relatedness supported this placement: OrthoANI with *R. cercidiphylli* IEGM 1322 was ~99%, and fastANI showed 98.9% to *R. cercidiphylli* and 97.1% to *R. cerastii*, but < 80% to *R. qingshengii* and *R. erythropolis* (Figure 1B; Table S3). Genome sizes were similar (R1B_2T = 5,497,800 bp; IEGM 1322 = 5,489,640 bp). Pangenome analysis across nine genomes revealed a small core (0.5%), with 14% shell and 17.4% cloud genes, indicating substantial accessory content and strain‐specific genes (Figure S1; Table S3). Together, these data support the designation of R1B_2T as a novel subspecies within the *R. cercidiphylli* lineage, potentially adapted to Arctic beach sediments. Consistent with this placement, hydrocarbon‐activation genes in R1B_2T (three *alkB* copies and an *almA*‐like monooxygenase) cluster with *R. cercidiphylli*/*cerastii* and are distinct from those of *R. erythropolis* and *R. qingshengii* (*alkB* clade bootstrap > 99%; Figure S2; Table S4).

### 3.2 Hydrocarbon Biodegradation Performance

**FIGURE 2:**
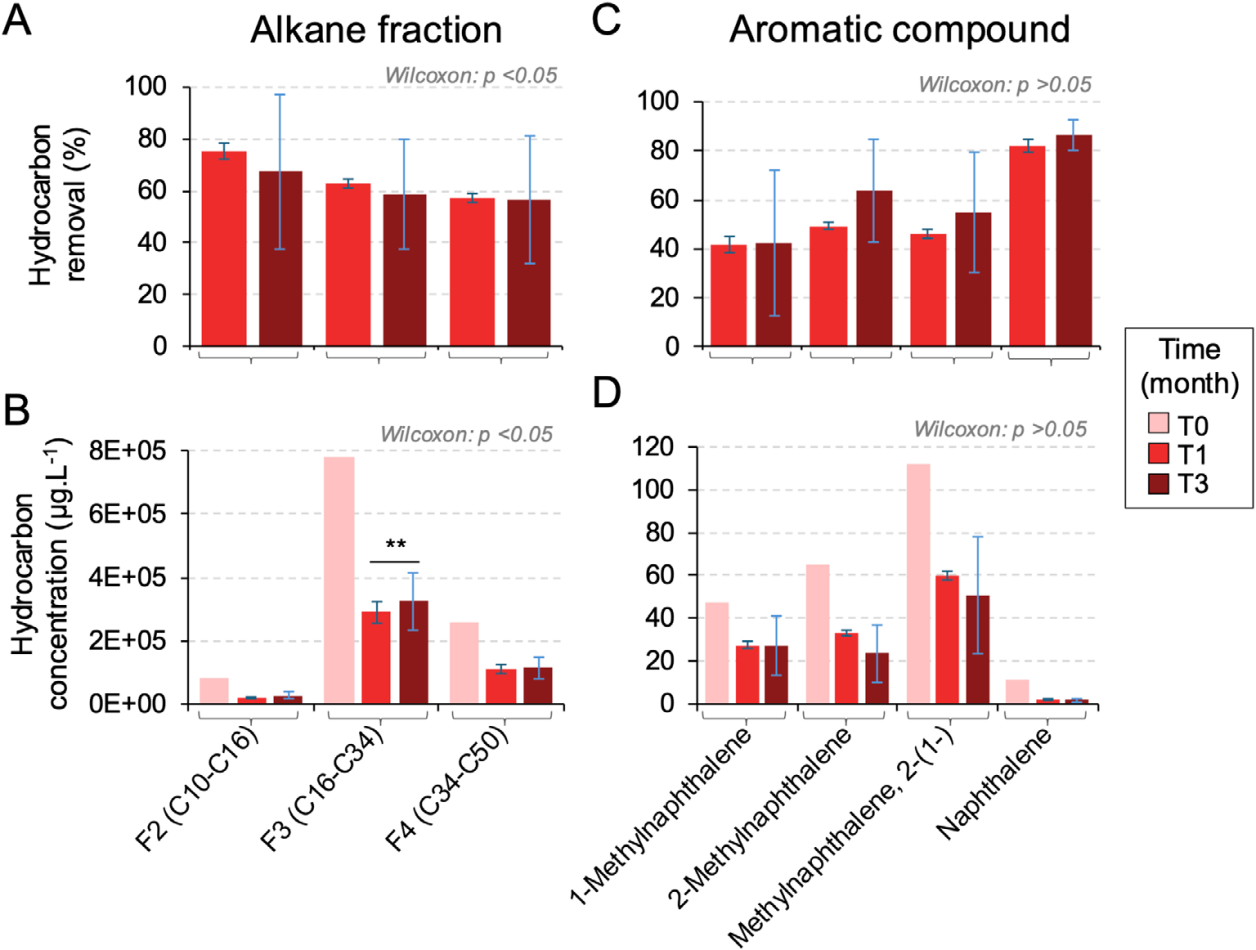
Petroleum hydrocarbon analyses of *Rhodococcus* exposed to ultra‐low sulphur fuel oil (ULSFO) over 3 months. (A) and (B) Alkane fractions (F2 to F4) degradation over time. (C) and (D) Aromatic compounds degradation over time. Error bars are for the triplicate cultures incubated for 1 and 3 months with biological replicates (*n* = 6). Missing error bars mean that triplicate values were not available. A complete list of values is in Table S1. Details of the One‐way ANOVA test for natural attenuation by *Rhodococcus* sp. strain R1B_2T over time are in Table S5.

Analysis of residual fuel after 1 and 3 months (T1 and T3) of exposure to *Rhodococcus* sp. strain R1B_2T showed similar percentages of ULSFO removal due to biodegradation at both time points (Figure 2, Table S1). For the alkane biodegradation, almost 80% of fraction 2 (C10–C16) was removed after a month (Figure 2A). The alkane fraction shows a clear and significant decrease (one‐way ANOVA, *p* value = 0.00171**; Table S5) between T1 and T3 of fuel exposure for only alkane fraction F3 (C16–C34; Figure 2B). For the aromatic compounds, the results are more varied (Figure 2C,D). The degradation of 1‐methylnaphthalene, 2‐methylnaphthalene, methylnaphthalene 2‐(1‐) and naphthalene showed no statistically significant differences between T1 and T3 (one‐way ANOVA, *p* value > 0.05; Table S5). Percent removals at T1 versus T3 were naphthalene 81.8% versus 86.4%, 1‐methylnaphthalene 41.5% versus 42.6%, 2‐methylnaphthalene 49.3% versus 64.0%, and methylnaphthalene 2‐(1‐) 46.4% versus 55.0%. Although the percent removal of naphthalene exceeded that of the methylated naphthalene, its initial concentration in ULSFO was lower (Figure 2D), tempering interpretation based on percentages alone. Overall, nonparametric comparisons indicated significant changes for alkane fractions from T1 to T3 (Wilcoxon test, *p* value < 0.05*), whereas no significant differences were detected for the aromatic compounds (*p* value > 0.05). Where relevant, percent removals for each fraction/compound at T1 and T3 are reported in Table S1 to contextualise these comparisons.

### 3.3 An Overview of Transcriptome Assembly and Annotation

**FIGURE 3:**
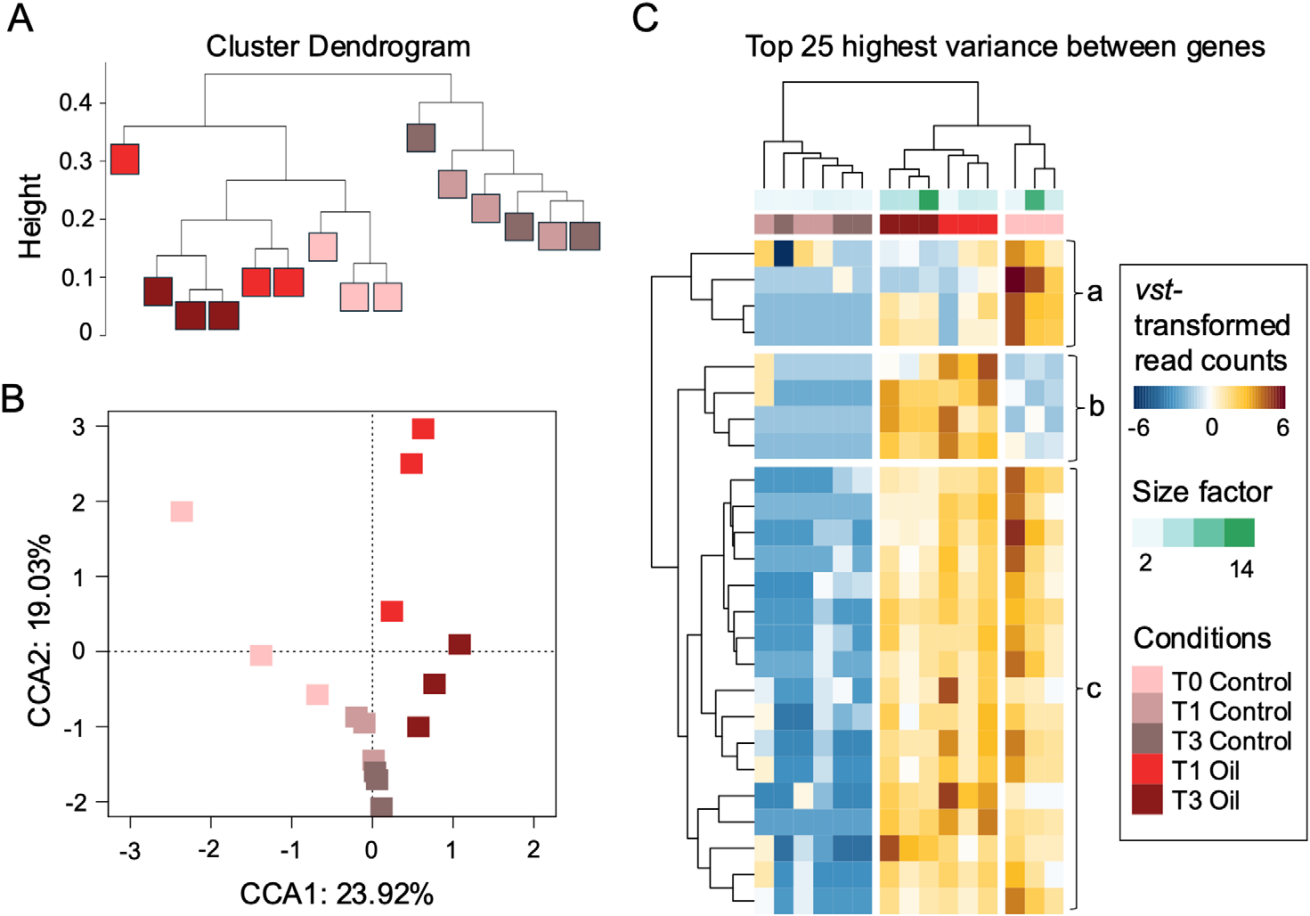
Clustering of transcriptome samples. (A) Hierarchical clustering of all samples based on normalised reads and unweighted pair group method with arithmetic mean (UPGMA) based on the Bray‐Curtis distance matrices of significant DEGs for all sampling times. (B) Canonical correspondence analysis (CCA) with the Bray–Curtis dissimilarity measure of reads per gene of all transcriptomes. (C) Top 25 differentially expressed genes of the *Rhodococcus* sp. strain R1B_2T. Heatmap of clustered results based on normalised and transformed read counts. The functional annotation of each gene is on the right of the heatmap, indicated letters a to c, refer to Table S6. The variance stabilising transformation (vst) is calculated with the *vst()* function within the *DeSeq2* package on R and transforms the count data to normalise the read count. The size factor corresponds to the sequencing depths of each transcriptome.

Differential gene expression from all 15 transcriptomes from triplicate conditions at timepoints showed consistent clustering patterns across three methods: a dendrogram (Figure 3A), a constrained correspondence analysis ordination (CCA; Figure 3B), and a heatmap of the 25 most variable genes (Figure 3C). The dendrogram showed distinct grouping of samples, with oil‐treated samples at T1 and T3 clustering separately from no‐oil controls. Similarly, the CCA separated samples by experimental condition, with control samples forming one cluster and oil‐treated samples another. The first two principal components accounted for 23.92% and 19.03% of the variance, respectively. The heatmap shows distinct expression patterns between the oil‐treated and control samples, with 4 genes in cluster b being upregulated in oil treatment samples, while the remaining 21 genes in clusters a and c (a: 4 genes; c: 17 genes) showed higher expression in control samples (Figure 3C). Within the 25 most variable genes in ULSFO‐exposed samples (T1, T3), we identified genes associated with fatty acid (FA) degradation, transport, and modification, including a cytochrome P450 (also known as *CYP153*) and *fadD* (K01897), a long‐chain acyl‐CoA synthetase; whereas most of the remaining genes were annotated under “Translation” and “Post‐translation” categories (Figure 3C, Table S6).

### 3.4 Differential Gene Expression Over Time Exposed to Oil

Throughout our analyses, we identified differentially expressed genes (DEGs), both up‐ and down‐regulated, during exposure to ULSFO. Control‐only comparisons (T0 vs. T1, T0 vs. T3, T1 vs. T3) showed relatively balanced expression changes, with similar numbers of up‐ and down‐regulated genes (Figure S3). In contrast, the comparison between control and oil treatments at the same time points (T1 Control vs. T1 Oil, T3 Control vs. T3 Oil) reveals substantial differences, including the highest numbers of DEGs (1219) at 1 month of exposure (T1 Oil vs. T1 Control). Oil treatments time series (T0 vs. T1 Oil, T0 vs. T3 Oil) also showed marked shifts, with the lowest number of DEGs (140) detected between T3 Oil and T1 Oil. The dot plots showed the significant DEGs (defined as DEGs with a *p* value < 0.05*), and this stringency reduced the number of DEGs considered (Figure S3).

### 3.5 Overall Functional Annotations

**FIGURE 4:**
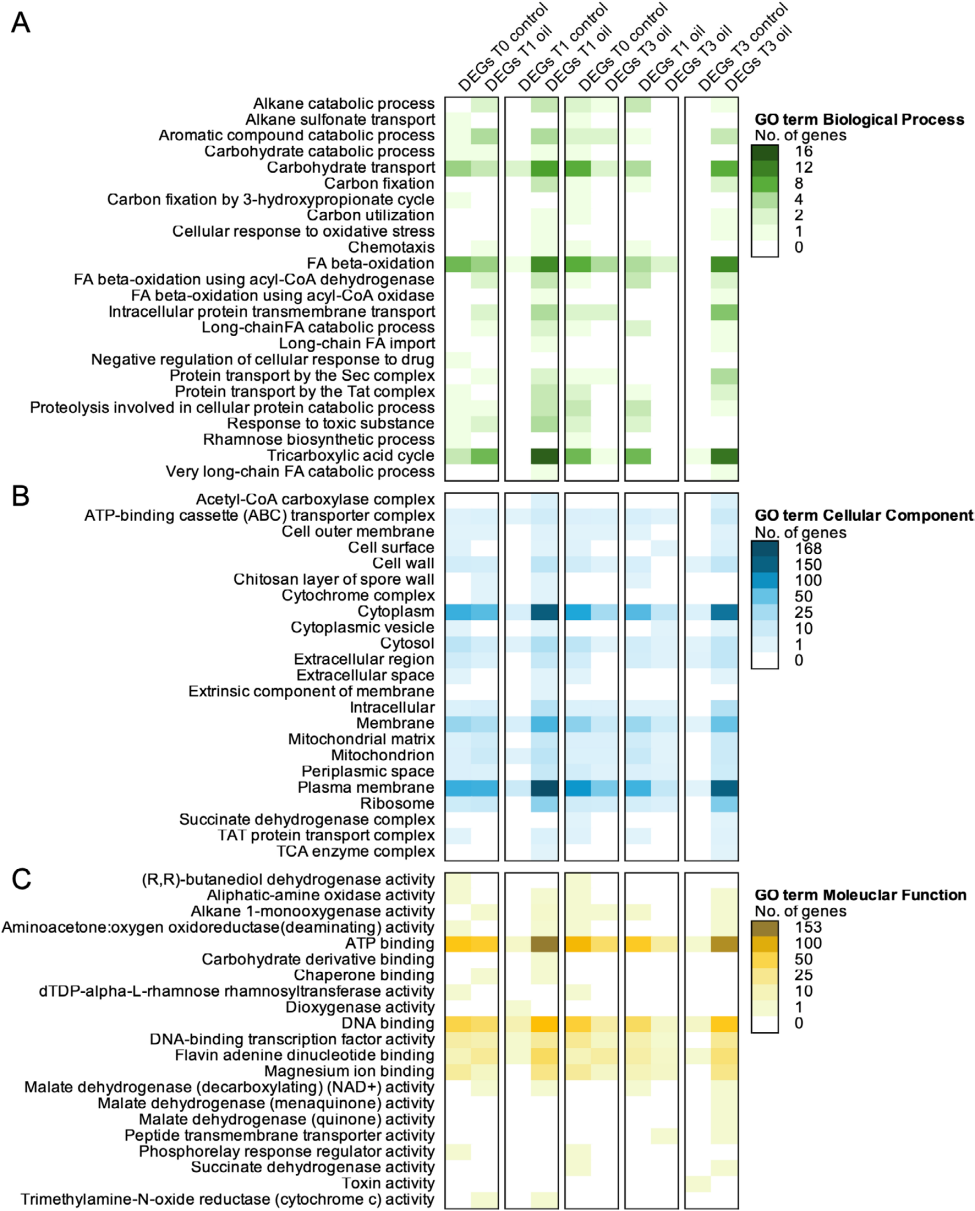
Top expression of differentially expressed genes (DEGs) annotated with gene ontology (GO) terms between comparisons of time and with or without ULSFO. (A) GO term of the biological process category. (B) GO term of the Cellular Component category. (C) GO terms of the Molecular Function category. A complete list of genes and annotations can be found in Table S7.

To further glean biological meaning from the DEGs resulting from the ULSFO exposure over time, we examined the placement of genes within the GO, COG and KEGG databases (Figures 4, S4, and S5). The GO terms provide functional annotation of gene products in three domains: Biological process (BP; Figure 4A), cellular component (CC; Figure 4B), and molecular function (MF; Figure 4C). In the BP domain, cells exposed to ULSFO at T1 and T3 had more DEGs annotated within three main processes: 16 genes involved in the tricarboxylic acid cycle (TCA; Table S7), 11 genes involved in FA beta‐oxidation, and 9 genes involved in carbohydrate transport. In the CC domain, a maximum of 168 and 154 DEGs encoded for ‘plasma membrane’ at T1 oil versus T1 control and at T3 oil versus T3 control, respectively (Figure 4B, Table S7). For the same comparison, we observed the highest number of DEGs encoding for ‘cytoplasm’. In the MF domain, a maximum of 153 DEGs were observed at T1 oil versus T1 control within the ‘ATP binding’.

Functional annotation from the COG database revealed two categories, where we observed the highest number of DEGs (5 genes at T1 Oil vs. T1 Control) encoding for an acyl‐CoA dehydrogenase related to the alkylation response protein AidB (COG1960) involved in FA biosynthesis; and ca. 4 DEGs encoding for an acyl‐CoA reductase related to aldehyde dehydrogenase (COG1012) involved in proline degradation (Figure S4, Table S8). At T3 Oil vs. T3 Control, we observed the highest number of DEGs (4 genes) within the category of FA desaturase (COG3239) and enoyl‐CoA hydratase/carnithine (COG1024) involved in FA biosynthesis. Within the KEGG database, in several pathways, we observed the overexpression of genes especially at T1 Oil vs. T1 Control, with 63 DEGs involved in FA metabolism (ko01212) and 56 DEGs involved in propanoate metabolism (ko00640; Figure S5, Table S9).

### 3.6 Co‐Expression Network of Differentially Expressed Genes

**FIGURE 5:**
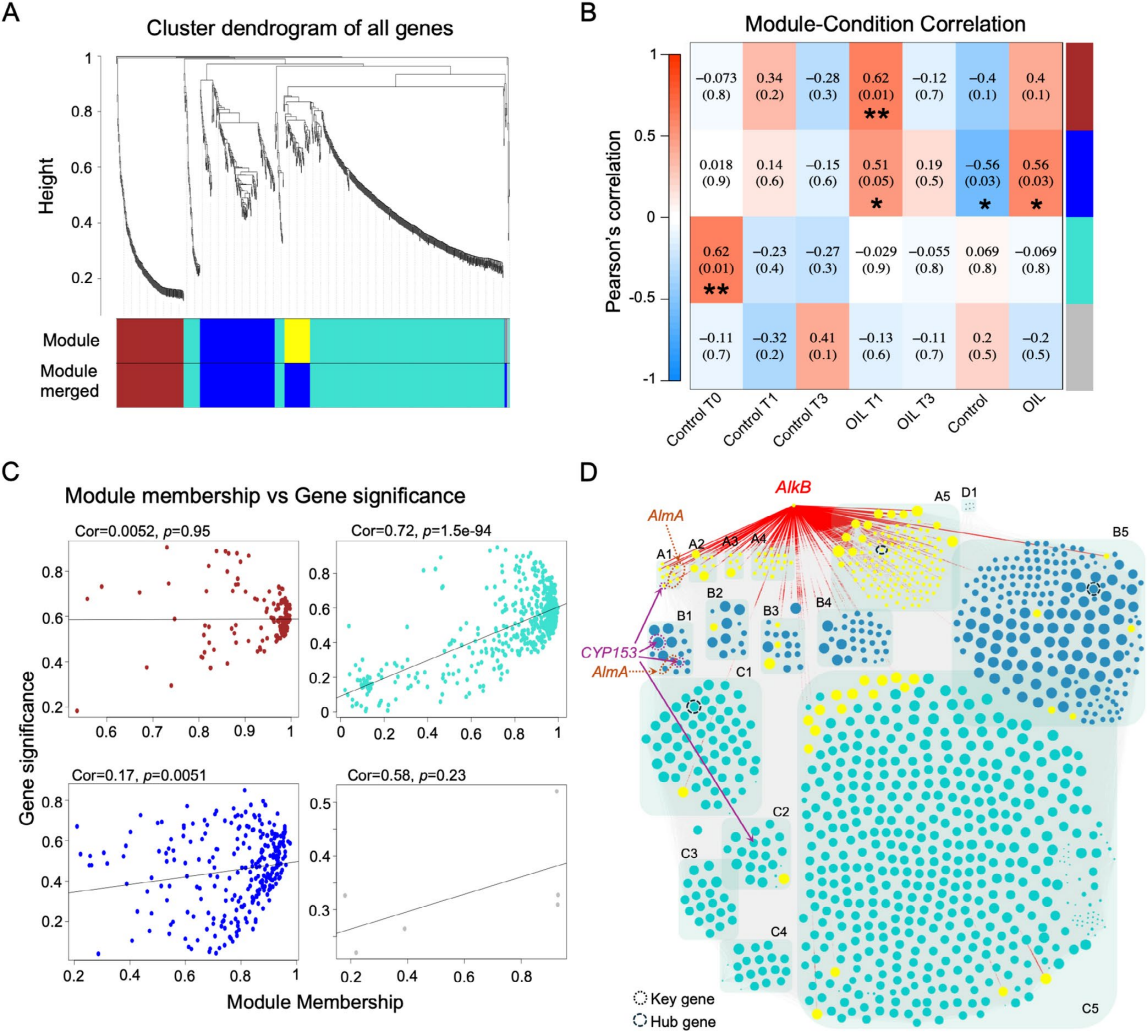
Weighted gene co‐expression network analysis (WGCNA) of differentially expressed genes (DEGs). (A) The dendrogram shows the DEGs that clustered into 8 modules. Genes were clustered based on a dissimilarity measure (1‐TOM). (B) Module with weighted correlations and corresponding *p* values at time of sampling, and salinity conditions. The colour scale shows module‐trait correlation based on Pearson's rank correlation. Asterisks within the correlation indicate the significance level *p* value from Pearson's *Rho* (*p* value < 0.05 *, < 0.01 ** and < 0.001 ***). (C) Scatter plots showing correlation between module membership and gene significance for four key modules (brown, blue, turquoise and grey). Each point represents a gene; trend lines show linear relationship; Cor = correlation coefficient, *p* = significance value. (D) Brown, turquoise and blue modules co‐expression network based on WGCNA analysis representing the significant DEGs interactions. The node size is calculated on the edge count. The higher value or bigger size node refers to a stronger connection or co‐expression of DEGs. Black dotted circles represent the hub gene in each module. Group of nodes from brown module are referred as A1 to A5, from blue module as B1 to B5, from turquoise module as C1 to C5 and from grey module as D1. Complete list of gene in each node and module can be found in Table S10.

We analysed 1037 DEGs across all transcriptomes using WGCNA, which grouped genes into four modules (Figure 5A). The dendrogram split into two main branches, with the brown module distinct from turquoise, blue, and grey. Module‐condition associations highlighted oil‐responsive programs (Figure 5B). The blue module correlated positively with oil treatments (both T1 and T3 Oil; *p* value < 0.05*), and negatively with controls. The brown module was specifically associated with T1 Oil condition (*p* value < 0.01**). The turquoise module correlated with T0 control (*p* value < 0.01**), indicating processes active under baseline conditions that are repressed during oil exposure. Gene significance–module membership relationships differed by module (Figure 5C): Turquoise showed a strong correlation (*r* = 0.72, *p* value = 1.5e‐94), blue a weaker but significant one (*r* = 0.17, *p* value = 0.005) and brown none (r = 0.005, *p* value = 0.95), suggesting complex regulation in brown where connectivity does not predict responsiveness. These patterns reveal distinct organisational principles in gene networks responding to hydrocarbon stress.

To investigate functional relationships between key hydrocarbon degradation genes, we constructed a focused network highlighting connections of *alkB*, *almA*, and *CYP153* genes within our co‐expression modules (Figure 5D). Across the 1037 nodes and 263,410 edges, *alkB* showed extensive connectivity within the oil‐associated brown module and links to blue and turquoise. Notably, brown module *alkB* connected to *CYP153* and *almA* within Brown but not to their counterparts in other modules, implying module‐specific coordination of degradation pathways. Each module featured distinct hub genes: the blue module's hub was a choline/glycine/proline betaine transport protein (*proV*, K02168) with 66 nodes and 790 connections; the brown module's hub was glycosyltransferase 4 (GT4), involved in cell wall biosynthesis, with 56 nodes and 694 connections; and the Turquoise module's hub was glycosyl hydrolase 13 subfamily 33 (GH13_33), functioning as a trehalose synthase and/or maltose glucosylmutase.

### 3.7 Differentially Expressed Genes of Specific Pathways

**FIGURE 6:**
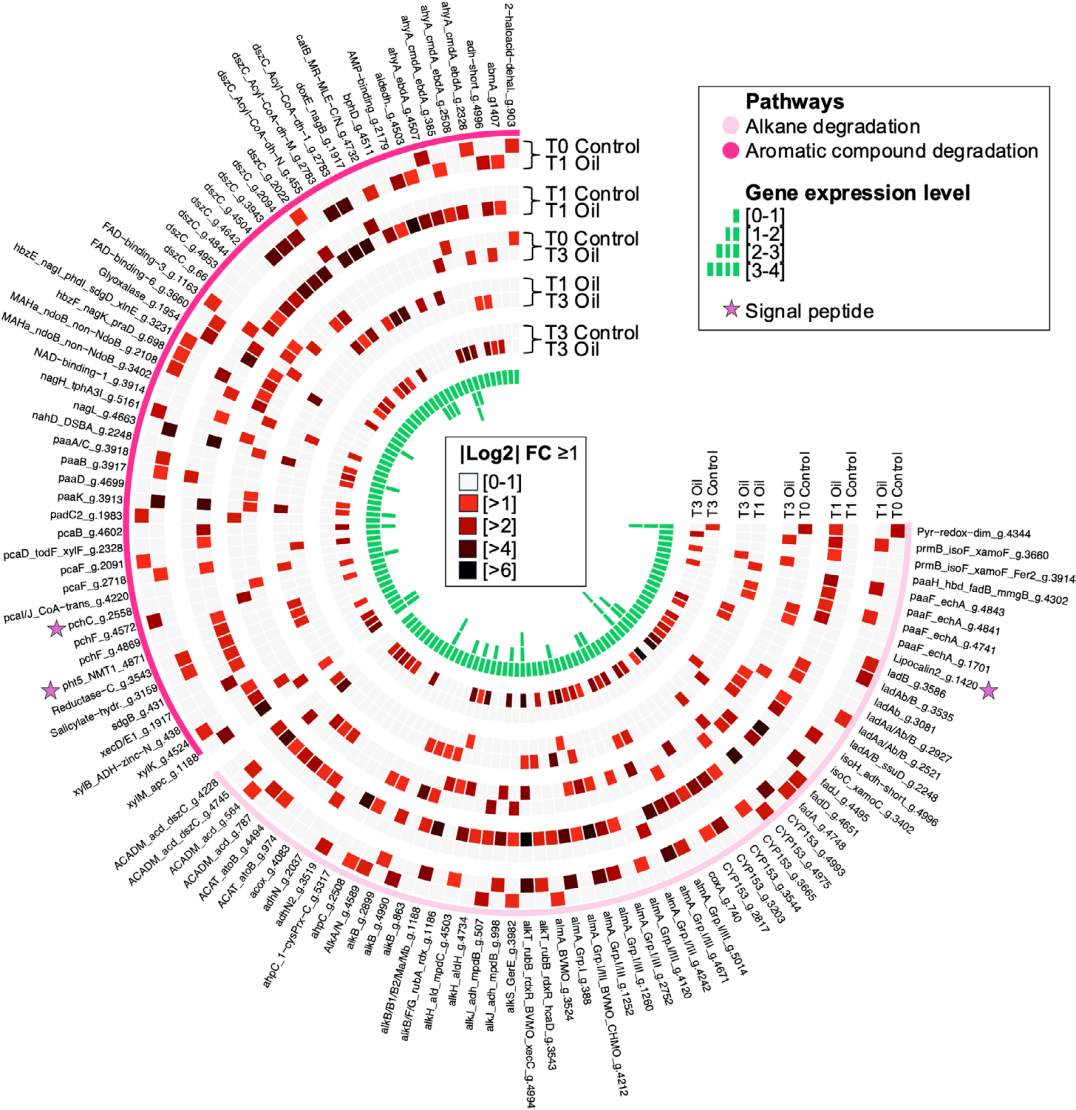
Circular heatmap of differentially expressed genes (DEGs) and their expression profile based on the log_2_ fold change between time of sampling (T0, T1—1 month, T3—3 months) and with or without ULSFO. Colours in the outer circle indicate alkane or aromatic compound degradation category for each DEG. The heatmap uses colour intensity to show the level of gene expression, with darker colours indicating higher expression levels. A complete list of genes and annotations can be found in Table S11.

**FIGURE 7:**
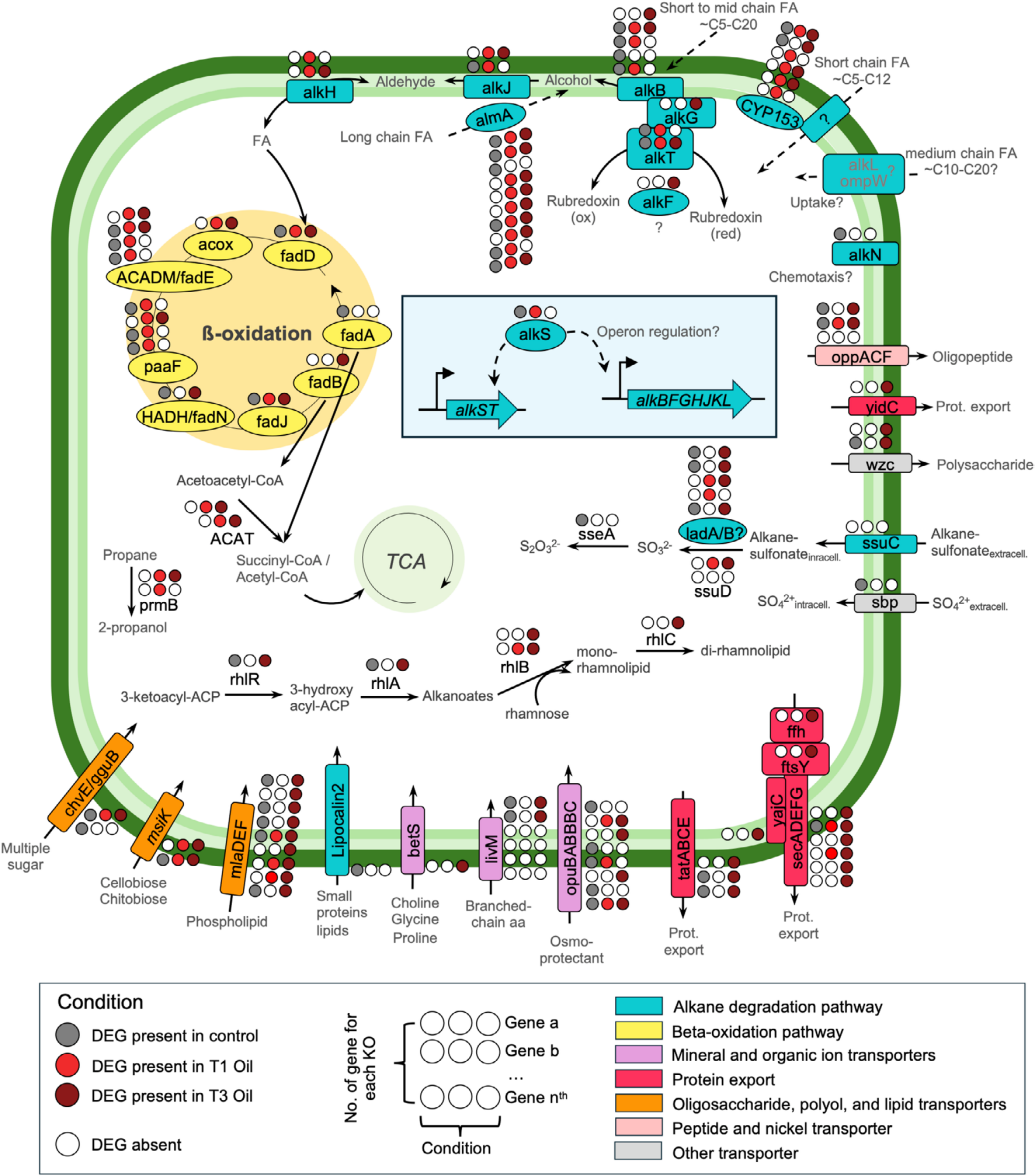
Key genes expressed in *Rhodococcus* for alkane compound degradation. A complete list of genes and annotations can be found in Tables S11 and S13.

**FIGURE 8:**
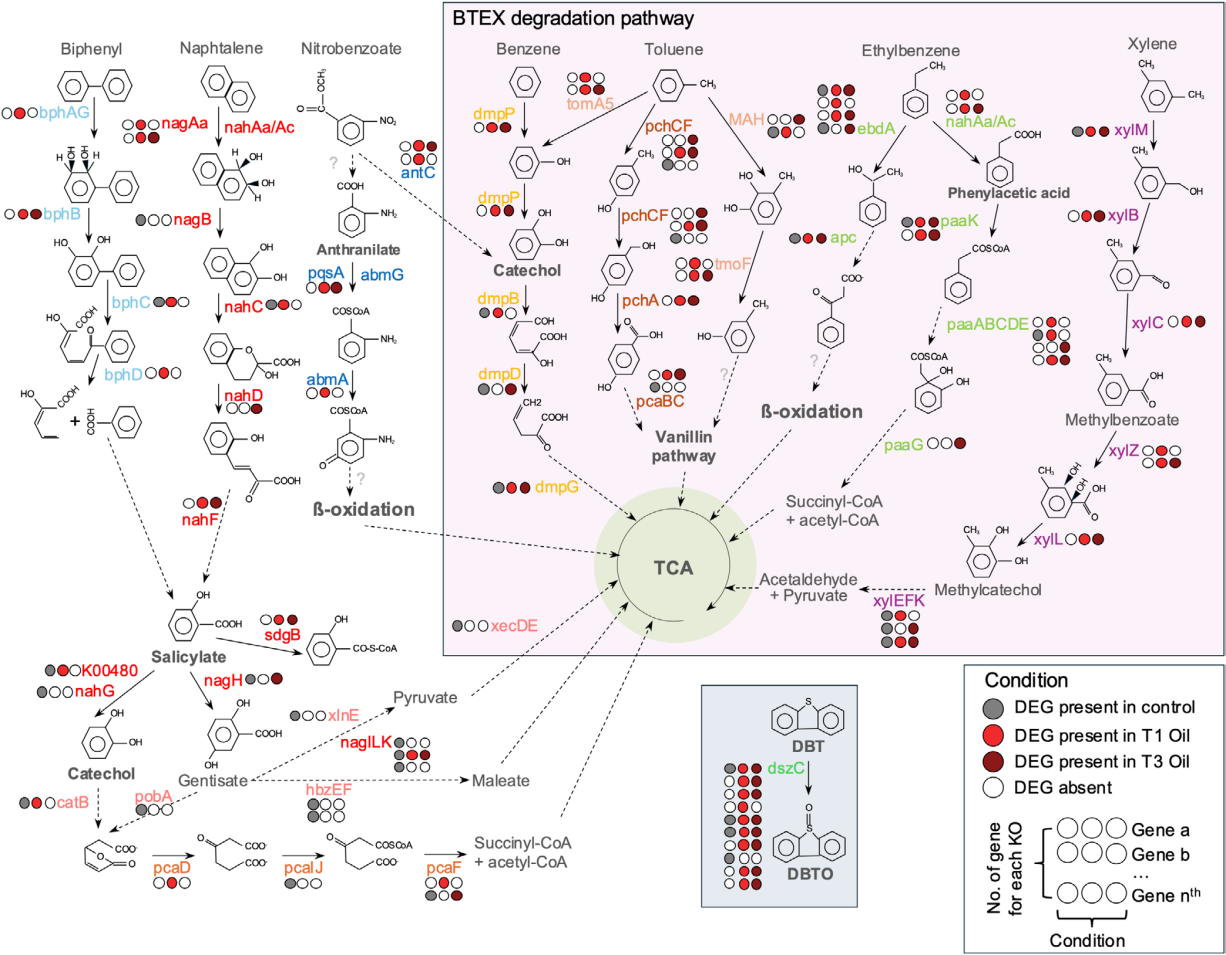
Key genes expressed in *Rhodococcus* for aromatic compound degradation. A complete list of genes and annotations can be found in Table S11.

We selected only DEGs that were identified to a specific annotation, or potential pathways involved in HD genes. This was achieved by leveraging the findings of module networks (Figure 5) and investigating functional annotations (Figures 4, 6, S4–S7). A schematic representation of the *Rhodococcus* cell is provided, offering a summary of the potential roles and implications of the selected DEGs in the response of the *Rhodococcus* exposed to ULSFO over a three‐month period (Figures 7 and 8). Several categories of pathways and key genes are further developed below.

### 3.8 Aliphatic and Aromatic Compounds Degradation by *Rhodococcus*

A total of 61 and 55 DEGs were identified coding for proteins involved in alkane and aromatic compound degradation pathways, respectively (Figures 6, 7, 8, Tables S11–S13). The most frequently detected protein involved in the alkane degradation pathway was the long‐chain alkane oxidising enzyme, *almA* group I and III, with or without the presence of the pfam domains BVMO (PF00743, PF07992 and PF13450) and/or CHMO (PF00743; Figure 7, Table S11). Of the ten DEGs coding for *almA*, some showed consistent expression across all conditions. However, five out of ten DEGs coding for *almA* displayed selective expression, appearing exclusively in either control samples (without ULSFO, 1 DEG) or those exposed to ULFSO (4 DEGs). Several DEGs in the alkane degradation pathway, such as rubredoxin proteins 1 (*alkF*, PF00301) and 2 (*alkG*, PF00301), and aldehyde dehydrogenase (*alkH*, PF00171), were expressed only in ULSFO conditions at T1, T3 or both.

In the aromatic compound degradation pathways, *Rhodococcus* strain R1B_2T exhibits DEGs encoding essential enzymes for the degradation of BTEX, biphenyl, naphthalene, nitrobenzoate and dibenzothiophene (DBT; Figure 8, Table S11). Notably, many DEGs coding for enzymes involved in the final steps of converting catechol into smaller molecules that enter the TCA cycle, and the TCA cycle were expressed in the control condition. These enzymes include salicylate hydroxylase (K00480), salicylate hydrolase *nahG* (K00480), which converts salicylate to catechol, and salicylate 5‐hydroxylase (*nagH*, PF13577), that converts salicylate to gentisate. Gentisate is further broken down into pyruvate via gentisate 1,2‐dioxygenase (*nagI*, Uniprot O86041), fumarylpyruvate hydrolase (*nagL*, O86042), maleylpyruvate isomerase (*nagK*, O86043), and *xlnE* (Q9S3U6), or to maleate via *hbzEF* (PF07883 and PF01557). In contrast, some DEGs were only expressed in response to ULSFO exposure. For instance, in the xylene degradation pathway, enzymes such as toluene methyl‐monooxygenase (*xylM*, K15757, PF00487), xylulokinase (*xylB*, K00854, PF00107, PF08240), benzaldehyde dehydrogenase (*xylC*, K00141), benzoate/toluate 1,2‐dioxygenase reductase component (*xylZ*, K05784) and dihydroxycyclohexadiene carboxylate dehydrogenase (*xylL*, K05783) were mainly up‐regulated at T1 and/or T3 incubated with ULSFO. Additionally, 10 DEGs coding for dibenzothiophene monooxygenase (*dszC*, K22219, PF02770, PF02771, PF08028), involved in converting DBT to DBT‐sulfoxide (DBTO), were identified. Among the 10 DEGs coding for *dszC*, varied expression patterns were displayed, with some being active in all conditions, while others were expressed exclusively in T1 and/or T3 under ULSFO exposure or control conditions.

### 3.9 Carbohydrate Active Enzymes Potentially Involved in Degradation

The analysis of carbohydrate‐active enzymes (CAZyme) (Cantarel et al. 2009) revealed 40 distinct CAZy families, with glycoside hydrolases (GHs) comprising 45% of the total, glycosyltransferases (GTs) making up 30%, auxiliary activities (AAs) accounting for 15%, carbohydrate esterases (CEs) contributing 7.5%, and carbohydrate‐binding modules (CBMs) representing 2.5%. Among the 74 DEGs identified as encoding CAZyme (Figure S6, Table S12), 17 were found to have a signal peptide. This signal peptide, an extension of the amino acid sequence, indicates where the protein is destined to be transported, whether inside or outside the cell (Briggs and Gierasch 1986). DEGs containing signal peptides were predominantly associated with the CE, CBM, and GH families. Several DEGs were exclusively expressed during the ULSFO exposure periods (1 and/or 3 months). These included 7 DEGs related to AA families (e.g., AA1, AA2, AA3, AA3_2, AA7), 2 DEGs encoding CBM48, 9 DEGs associated with CE families (e.g., ce1, ce5, ce14), 12 DEGs encoding GH families (e.g., GH3, GH13_3, GH13_10, GH13_11, GH13_20, GH15, GH43_23, GH77), and 15 DEGs related to GT families (e.g., GT2, GT2_3, GT4, GT20, GT35, GT39, GT51, GT87, GT89).

### 3.10 Key Enabling Processes and Genes for Cold‐Environment Oil Degradation

We use ‘key enabling processes and genes’ to refer to functions that facilitate hydrocarbon uptake and metabolism under cold conditions, such as biosurfactant production, hydrocarbon activation/transport, polymer/complex‐organic degradation, cold‐shock/chaperone responses, protein export/secretion, and osmoprotection. Guided by this framework, HADEG‐based analyses (Rojas‐Vargas et al. 2023) identified DEGs across these categories (Figure S7, Table S13). Of the 78 DEGs, 8 contained signal peptides linked to osmoprotection, polymer degradation, and transporter functions. The final steps of rhamnolipid biosynthesis in *Rhodococcus* were exclusively expressed under ULSFO conditions (T1 and T3 Oil; Figures 7 and S7), involving helicase domain proteins (PF00270, PF00271 in *rhlB*; PF13641 in *rhlC*). Additionally, two DEGs expressed in control and T3 Oil encoded an HTH‐type quorum‐sensing regulator (*rhlR/gerE*, PF00196, PF03472) and a 3‐(3‐hydroxydecanoyloxy) decanoate synthase (*rhlA*, PF00561). Twenty DEGs related to polymer degradation were found, 11 of which were exclusive to ULSFO exposure. Two *cspA* DEGs (K03704) were upregulated in T3 Oil, and two *groEL* DEGs (K04077) were expressed in T3 Oil, with one also in the control condition.

For osmoprotection, 8 DEGs were identified in the osmoprotectant transporter system: 3 encoding the ATP‐binding protein *opuBA* (K05847), 2 for the permease protein *opuBB* (K05846), and 2 with signal peptides for the substrate‐binding protein *opuBC* (K05845). One DEG encoded *opuBC/AC* (K05845, PF04069) with a substrate‐binding domain for the ABC‐type glycine betaine transport system (Figures 7 and S7). Four DEGs (*opuBA* g.4676, *opuBB* g.4677, *opuBC* g.4674, g.4675) formed a cluster, exclusively expressed under ULSFO conditions. All 14 DEGs related to secretion and protein export were also expressed under ULSFO (Figures 7 and S7), including a membrane protein insertase *yidC* (K03217, PF02096), the preprotein translocase complex *yajC (*K03210), *secADEFG* (K03070, K03072, K12257, K03073, K03074 and K03075), and sec‐independent translocases *tatA/E* (K03116), *tatB* (K03117) and *tatC* (K03118). Six DEGs encoding cytochrome p450, involved in transporting FA (C10‐C20), were found, but no DEGs for short‐chain FA transporters like *alkL* or *ompW* were detected.

## 4 Discussion

To adapt to external changes or environmental stressors, cells adjust their transcriptome to maintain internal stability (López‐Maury et al. 2008). Here, we define stress as the exposure to ULSFO, a substantial environmental challenge requiring metabolic adaptation. If such a perturbation persists, cells can acclimate to the new conditions through transcriptional adjustments and continue growing (Kültz 2005). With declining sea ice in the NWP, ice‐influenced coastal Arctic environments face increased oil spill risk from shipping. Therefore, we simulated a petroleum marine environment, exposing an Arctic coastal marine beach sediment isolate, *Rhodococcus* sp. strain R1B_2T, to ULSFO for 3 months.

Our results converge on three main insights: (i) a sequential degradation strategy that prioritises alkanes before aromatics, (ii) timed rather than continuous expression of multiple hydrocarbon degradation pathways, and (iii) a versatile metabolic toolkit that extends from central metabolism to biosurfactant production. Together, these traits point to evolved mechanisms for surviving and metabolising oil under cold, oligotrophic Arctic conditions.

### 4.1 Sequential Degradation as an Energy‐Conservation Strategy in Cold Environments

*Rhodococcus* sp. strain R1B_2T metabolised alkanes rapidly in the early phase of exposure, followed by increased degradation of aromatics at later timepoints (Figure 2). This pattern reflects the preferential use of simpler aliphatics before shifting to more complex aromatics, a strategy consistent with previous observations in hydrocarbon‐degrading communities (Van Hamme et al. 2003; Bento et al. 2005; Coulon et al. 2005). The modest changes in naphthalene and its derivatives compared to the faster loss of alkanes further support this substrate hierarchy, which likely also reflects the low initial PAH content of ULSFO (Yang et al. 2023). Gene expression dynamics mirrored these shifts, with early activation of pathways for alkane oxidation and fatty acid metabolism, followed by upregulation of aromatic catabolism and functions related to biosurfactant production and organic matter turnover (Figures 6, 7, 8). Such temporal transitions suggest an adaptive strategy in which initial stress responses and rapid energy acquisition give way to improved access and processing of more recalcitrant fractions.

Similar trajectories have been reported for other cold‐adapted hydrocarbon degraders, including *Alcanivorax*, *Cycloclasticus* and *Oleispira* (Leahy and Colwell 1990; Mohn and Tiedje 1992; Head et al. 2006; Schneiker et al. 2006; Teira et al. 2007; Kube et al. 2013), underscoring the ecological relevance of this phased degradation strategy. This pattern contrasts with temperate *Rhodococcus* strains, where degradation genes are often activated simultaneously (Whyte et al. 2002). It also extends beyond the specialisation seen in psychrophiles such as *Oleispira antarctica* , which primarily target alkanes (Kube et al. 2013) and rivals versatile heterotrophs such as *Colwellia psychrerythraea* 34H (Methé et al. 2005). Together, these comparisons support the idea that R1B_2T's sequential strategy represents a cold‐environment innovation facilitating persistence in Arctic oil‐impacted habitats.

Importantly, we observed sequential activation and apparent coordination among *alkB*, *CYP153*, and *almA*, suggesting a regulatory mechanism that staggers the use of overlapping pathways (Figure 5D), an attribute not previously reported in cold‐adapted *Rhodococcus* strains such as *R. qingshengii* (Lincoln et al. 2015) and *R. erythropolis* (Laczi et al. 2015). This synergy between *alkB*, *CYP153* and *almA* likely represents a complementary degradation strategy where *alkB* preferentially oxidises short to medium‐chain alkanes (~C5–C20) and > C30 with other functional enzymes (Fenibo et al. 2023; Suzuki et al. 2025), while CYP153 more efficiently targets shorter chains (~C5–C12) with some substrate range overlap (van Beilen et al. 2006; Fenibo et al. 2023; Suzuki et al. 2025). CYP153 is thought to cooperate with *alkB* in n‐alkanes oxidation (Nikaido 2003; Liang et al. 2016). As for flavin‐binding alkane hydroxylase *almA* and flavin‐dependent alkane monooxygenase *ladA*, both oxidise long‐chain alkanes, C28–C36 and C10–C30, respectively (Xiang et al. 2023; Suzuki et al. 2025), indicating broad chain‐length coverage.

### 4.2 Timed Gene Regulation and Metabolic Coordination

A striking feature of R1B_2T is the precise timing of hydrocarbon degradation genes. For example, *alkS* was expressed only at control and one‐month timepoints (Figures 6 and 7), rather than continuously, indicating a regulated switch rather than constitutive activation. This suggests that R1B_2T avoids unnecessary metabolic costs by activating pathways only when needed, conserving energy in oligotrophic conditions. Consistent with reports that *Rhodococcus* harbours multiple *alkB* operons (Whyte et al. 2002), strain R1B_2T encodes several alkane‐degradation pathways, including *alkB*, *ladA* and *almA* (Figure 7), highlighting its potential for effective application in oil‐contaminated environments, especially under cold and saline conditions, since this strain is a halotolerant psychrophile (Lirette et al. 2024).

Central carbon metabolism was actively rerouted during exposure. Upregulation of dehydrogenases, β‐oxidation enzymes, and TCA cycle genes (Figures 4, S4, and S5) indicates efficient funnelling of hydrocarbon‐derived carbons into respiration. Enhanced oxidative phosphorylation (Figures 4, 7, and 8) suggests that this metabolism is energy‐yielding and tightly coupled to growth under oil stress (Atlas 1981; Margesin and Schinner 2001).

Membrane remodelling and stress responses also supported adaptation. Upregulation of cold shock proteins, chaperones, osmoprotectant transporters, and lipid biosynthesis genes (Figures S4 and S7) indicates a cellular strategy to maintain integrity and function in the presence of hydrophobic compounds. In this context, these physiological responses are not simply generic stress markers (Choudhary et al. 2024) but integrated components of an adaptive hydrocarbon degradation program in the Arctic. We also observed increased activity in fatty acid biosynthesis, elongation, and metabolism (Figure S5), consistent with lipid remodelling for membrane adaptation and utilisation of aliphatic substrates as carbon sources (Leahy and Colwell 1990; Mohn and Tiedje 1992).

CAZymes further expanded degradative capacity. Rather than enumerating each GH/CE family, we note that their upregulation at later stages coincided with the switch from alkanes to more complex substrates (Figure S6). This suggests enzymatic flexibility enabling utilisation of harder‐to‐access hydrocarbons and associated organic matter, sustaining metabolism after the simpler fraction is depleted. Multiple hydrocarbon degraders deploy CAZymes such as *Pseudomonas putida* and *P. aeruginosa*, which encode diverse GH and CE enzymes linked to carbohydrate and hydrocarbon processing (Margesin and Schinner 2001). CAZymes are also reported in *Marinobacter* (Gauthier et al. 1992), *Acinetobacter baumannii* (Towner 1992), *Bacillus subtilis* (Nimrat et al. 2019), and *Dietzia* (formerly *Rhodococcus*), with roles in alkane degradation and biosurfactants (Rainey et al. 1995; Soltanighias et al. 2019).

### 4.3 Metabolic Versatility and Bioremediation Potential

Beyond sequential use and timed regulation, R1B_2T exhibits a broad metabolic toolkit. This includes biosurfactant production, diverse transporters, and secondary metabolite systems that collectively improve hydrocarbon accessibility and detoxification. Notably, biosurfactant‐related genes coding, for example, acyltransferases and LuxR regulators (Figures 7 and S7, Table S13), were induced after oil exposure, enhancing emulsification and availability of hydrophobic substrates (Desai and Banat 1997; Mulligan 2005; Kothari and Jobanputra 2022). In *Rhodococcus* sp. strain R1B_2T, biosurfactant production likely reduces the size of large oil droplets, increasing surface area for enzymatic action. Biosurfactants, like rhamnolipids, are well‐known in hydrocarbon biodegraders, especially in high G + C content bacteria, like *Rhodococcus* species (Whyte et al. 1997; Raymond‐Bouchard et al. 2018). Their biodegradable nature makes them environmentally favourable while persisting long enough to support oil degradation effectively. Previous studies on *Rhodococcus* species support these traits (Whyte et al. 1997; Pal et al. 2009; Pacheco et al. 2010; Raymond‐Bouchard et al. 2018), consistent with bioremediation potential in cold marine systems. In Arctic *Rhodococcus*, biosurfactant production likely reduces oil droplet size, increasing surface area for enzymatic action. Combined with cold tolerance and biofilm formation, these traits strengthen its role in hydrocarbon degradation in marine systems (Perfumo et al. 2010).

Such versatility parallels but extends the strategies reported for *Alcanivorax borkumensis* and *Pseudomonas putida* and *P. aeruginosa* , where biosurfactants are central to hydrocarbon uptake (Yakimov et al. 1998; Margesin and Schinner 2001). In R1B_2T, combining biosurfactant synthesis with cold tolerance, stress proteins, and multi‐pathway hydrocarbon oxidation creates an unusually robust system. These traits collectively position R1B_2T as an effective degrader of complex oil mixtures under Arctic conditions. Compared with temperate *Rhodococcus*, its unique combination of sequential strategy, timed regulation, and metabolic breadth likely reflects adaptation to the fluctuating and mixed hydrocarbon inputs of the NWP. From a practical perspective, these findings highlight the strain's potential application in Arctic‐specific bioremediation strategies, where physiological resilience and metabolic flexibility are critical.

## 5 Conclusion

Our study reveals key novel insights into cold‐adapted hydrocarbon degradation mechanisms. Unlike mesophilic *Rhodococcus* strains, our Arctic isolate demonstrates a distinctive sequential degradation strategy, metabolising alkanes during early exposure before shifting to aromatic compounds later. This temporal orchestration represents a specialised adaptation to conserve energy in cold, oligotrophic conditions. Our transcriptomic analysis identified novel timed expression patterns of hydrocarbon degradation genes, including *alkS*, that are absent in mesophilic strains. The co‐expression network analysis revealed novel functional relationships between *alkB*, *CYP153*, and *almA* pathways that differ from the independent operation typically observed in mesophilic strains. When compared with other psychrotolerant hydrocarbon degraders, our *Rhodococcus* strain exhibits a more versatile metabolic toolkit for degrading multiple hydrocarbon classes, suggesting an evolved adaptation to the variable hydrocarbon mixtures in coastal Arctic environments. Together, these findings advance our understanding of cold‐adapted bioremediation mechanisms and provide valuable insights for developing Arctic‐specific remediation technologies for the increasingly vulnerable NWP.

## Author Contributions

**Nastasia J. Freyria:** data curation, formal analysis, visualization, investigation, methodology, project administration, writing – original draft, writing – review and editing, validation, software. **Antoine‐Olivier Lirette:** conceptualization, methodology, writing – review and editing. **Brady R. W. O'Connor:** data curation, writing – review and editing. **Charles W. Greer:** resources, supervision, writing – review and editing. **Lyle G. Whyte:** writing – review and editing, resources, funding acquisition, supervision, conceptualization, project administration, validation.

## Conflicts of Interest

The authors declare no conflicts of interest.

## Supporting information


**Table S1:** (xlsx). Petroleum hydrocarbon initial concentration, concentration measured, concentration of degradation and percentage of hydrocarbon removal from Figure 2.
**Table S2:** (xlsx). Overall summary of results of the Illumina transcriptome sequencing.
**Table S3:** (xlsx). Genome information and average nucleotide identity values between selected reference genomes of genus Rhodococcus from the pangenome comparison from Figures 1 and S1.
**Table S4:** (xlsx). Blastp results from comparison of almA gene annotated as putative flavin‐binding monooxygenase from Alloalcanivorax dieselolei (ADP30851.1) with 8 referenced genomes of Rhodococcus species and novel strain R1B_2T.
**Table S5:** (xlsx). Statistic one‐way ANOVA for total petroleum hydrocarbon analyses from Figure 2.
**Table S6:** (xlsx). List of genes and functional annotation from clusters of top 25 heatmap from Figure 3.
**Table S7:** (xlsx). List of Gene Ontology (GO) terms from Figure 4.
**Table S8:** (xlsx). List of Clusters of Orthologous Genes (COG) terms from Figure S4.
**Table S9:** (xlsx). List of KEGG eukaryotic Ortholog Groups of proteins (KO) terms from Figure S5.
**Table S10:** (xlsx). List of genes present in each module and group of nodes from Figure 5D.
**Table S11:** (xlsx). List of hydrocarbon degradation genes from Figures 6–8.
**Table S12:** (xlsx). List of genes coding for CAZY enzymes from Figure S6.
**Table S13:** (xlsx). List of genes from additional categories from Figures 7 and S7.

**Figure S1:** Pangenome of 10 *Rhodococcus* species. The circular pangenome compares 9 genomes of referenced species of *Rhodococcus* with *Rhodococcus*. sp. strain R1B_2T. Core gene: genes present in all genomes; cloud gene: genes present in less than 15% of genomes; shell gene: genes present in 15%–95% of genomes; and unique gene: genes present in only 1 genome. Genomes information can be found in Table S3.
**Figure S2:** A Maximum Likelihood alkB and almA genes trees generated using RAxML. (A) alkB genes phylogenetic tree constructed with 31 nucleic acid sequences of 1390 bp aligned along with 8 genomes of different species of Rhodococcus and with novel strain R1B_2T. (B) almA genes phylogenetic tree constructed with 34 amino acid sequences of 560 aa aligned along with 8 genomes of different species of Rhodococcus and with novel strain R1B_2T. The trees were constructed with bootstrap support calculated over 1000 repetitions. Only bootstrap values greater than 50 (out of 100) are displayed. Blastp results for almA genes can be found in Table S4.
**Figure S3:** Differential expression genes (DEGs) between time of sampling (T0, T1 and T3) with and without oil. Scatter plots show the comparison between months (T, T1–1 month; T3–3 months) of cells exposed either with ultra‐low sulphur fuel oil (ULSFO) or without oil (control). Each point represents a unigene. Points greater than 1 of log2 Fold Change (FC) indicate up‐regulated genes and lower than −1 indicate down‐regulated genes. Colour points indicate differential expressed genes (DEGs). Symbol “+” indicate only filtered DEGs with an Benjamini‐Hochberg‐adjusted *p* value < 0.05.
**Figure S4:** Top expression of differentially expressed genes (DEGs) annotated with Clusters of Orthologous Genes (COG) between comparisons of time and with or without the presence of ULSFO. A complete list of genes and annotations can be found in Table S8.
**Figure S5:** Top expression of differentially expressed genes (DEGs) annotated with KEGG Orthology (KO) between comparisons of time and with or without the presence of ultra‐low sulphur fuel oil (ULSFO). A complete list of genes and annotations can be found in Table S9.
**Figure S6:** Circular heatmap of differentially expressed genes (DEGs) annotated as carbohydrate‐active enzymes (CAZy) categories. The heatmap depicts expression profiles based on log2 fold changes across sampling timepoints (T0, T1–1 month, T3–3 months) under conditions with and without Ultra‐Low Sulphur Fuel Oil (ULSFO). The outer circle colour‐codes each DEG according to its specific CAZy category. Expression intensity is represented by colour gradient, with darker shades indicating higher expression levels. Table S12 provides a comprehensive list of all genes with their annotations.
**Figure S7:** Circular heatmap of differentially expressed genes (DEGs) annotated as different pathway categories. The heatmap depicts expression profiles based on log2 fold changes across sampling timepoints (T0, T1–1 month, T3–3 months) under conditions with and without ultra‐low sulphur fuel oil (ULSFO). The outer circle colour‐codes each DEG according to its specific pathway annotation category. Expression intensity is represented by colour gradient, with darker shades indicating higher expression levels. Table S13 provides a comprehensive list of all genes with their annotations.

## Data Availability

The raw transcriptome sequencing datasets generated in this study are available at NCBI under BioProject ID PRJNA1193011. Whole genome sequencing is available on the JGI genome portal under Gold Sequencing Project ID Ga0632451, Gold Analysis Project ID Ga0557218, and raw sequencing reads are available at NCBI under BioProject ID PRJNA945214.
