# Supplementary material for: Transcriptomic Analyses Unveil Hydrocarbon Degradation Mechanisms in a Novel Polar Rhodococcus sp. Strain R1B_2T From a High Arctic Intertidal Zone Exposed to Ultra‐Low Sulphur Fuel Oil
Source: Environ Microbiol Rep. 2025 Oct 16;17(5):e70218. doi: 10.1111/1758-2229.70218 (PMC12530890; doi:10.1111/1758-2229.70218)
Supplement: Supplementary file 2 — Figure S1: Pangenome of 10 Rhodococcus species. The circular pangenome compares 9 genomes of referenced species of Rhodococcus with Rhodococcus. sp. strain R1B_2T. Core gene: genes present in all genomes; cloud gene: genes present in less than 15% of genomes; shell gene: genes present in 15%–95% of genomes; and unique gene: genes present in only 1 genome. Genomes information can be found in Table S3. Figure S2: A Maximum Likelihood alkB and almA genes trees generated using RAxML. (A) alkB genes phylogenetic tree constructed with 31 nucleic acid sequences of 1390 bp aligned along with 8 genomes of different species of Rhodococcus and with novel strain R1B_2T. (B) almA genes phylogenetic tree constructed with 34 amino acid sequences of 560 aa aligned along with 8 genomes of different species of Rhodococcus and with novel strain R1B_2T. The trees were constructed with bootstrap support calculated over 1000 repetitions. Only bootstrap values greater than 50 (out of 100) are displayed. Blastp results for almA genes can be found in Table S4. Figure S3: Differential expression genes (DEGs) between time of sampling (T0, T1 and T3) with and without oil. Scatter plots show the comparison between months (T, T1–1 month; T3–3 months) of cells exposed either with ultra‐low sulphur fuel oil (ULSFO) or without oil (control). Each point represents a unigene. Points greater than 1 of log2 Fold Change (FC) indicate up‐regulated genes and lower than −1 indicate down‐regulated genes. Colour points indicate differential expressed genes (DEGs). Symbol “+” indicate only filtered DEGs with an Benjamini‐Hochberg‐adjusted p value < 0.05. Figure S4: Top expression of differentially expressed genes (DEGs) annotated with Clusters of Orthologous Genes (COG) between comparisons of time and with or without the presence of ULSFO. A complete list of genes and annotations can be found in Table S8. Figure S5: Top expression of differentially expressed genes (DEGs) annotated with KEGG Orthology (KO) bet [file EMI4-17-e70218-s001.docx]

**Transcriptomic analyses unveil hydrocarbon degradation mechanisms in a newly isolated polar *Rhodococcus* sp. strain R1B_2T from a high Arctic intertidal zone exposed to ultra-low sulfur fuel oil.**

**Nastasia J. Freyria^1*^, Antoine-Olivier Lirette^1,2^, Brady R.W. O’Connor^1^, Charles W. Greer^1,3^ & Lyle G. Whyte^1^**

^1^Department of Natural Resource Sciences, Faculty of Agricultural and Environmental Sciences, McGill University, Ste. Anne-de-Bellevue, Quebec, H9X 3V9, Canada

^2^ Graduate School of Agriculture, Hokkaido University, Sapporo 060-8589, Japan

^3^Energy, Mining and Environment Research Centre, National Research Council of Canada, 6100 Royalmount Ave., Montreal, QC, H4P 2R2, Canada

*** Correspondence:**

Corresponding Author

[nastasia.freyria@mcgill.ca](mailto:nastasia.freyria@mcgill.ca)

**Table S1 (xlsx).** Petroleum hydrocarbon initial concentration, concentration measured, concentration of degradation and percentage of hydrocarbon removal from **Fig. 2.**

**Table S2 (xlsx).** Overall summary of results of the Illumina transcriptome sequencing.

**Table S3 (xlsx).** Genome information and average nucleotide identity values between selected reference genomes of genus *Rhodococcus* from the pangenome comparison from **Figs. 1 and S1.**

**Table S4 (xlsx).** Blastp results from comparison of *almA* gene annotated as putative flavin-binding monooxygenase from *Alloalcanivorax* *dieselolei* (ADP30851.1) with 8 referenced genomes of *Rhodococcus* species and novel strain R1B_2T.

**Table S5 (xlsx).** Statistic one-way ANOVA for total petroleum hydrocarbon analyses from **Fig. 2.**

**Table S6 (xlsx).** List of genes and functional annotation from clusters of top 25 heatmap from **Fig. 3.**

**Table S7 (xlsx).** List of Gene Ontology (GO) terms from **Fig. 4.**

**Table S8 (xlsx).** List of Clusters of Orthologous Genes (COG) terms from **Fig. S4.**

**Table S9 (xlsx).** List of KEGG eukaryotic Ortholog Groups of proteins (KO) terms from **Fig. S5.**

**Table S10 (xlsx).** List of genes present in each module and group of nodes from **Fig. 5D.**

**Table S11 (xlsx).** List of hydrocarbon degradation genes from **Figs. 6-8.**

**Table S12 (xlsx).** List of genes coding for CAZY enzymes from **Fig. S6.**

**Table S13 (xlsx).** List of genes from additional categories from **Figs. 7 and S7.**

**
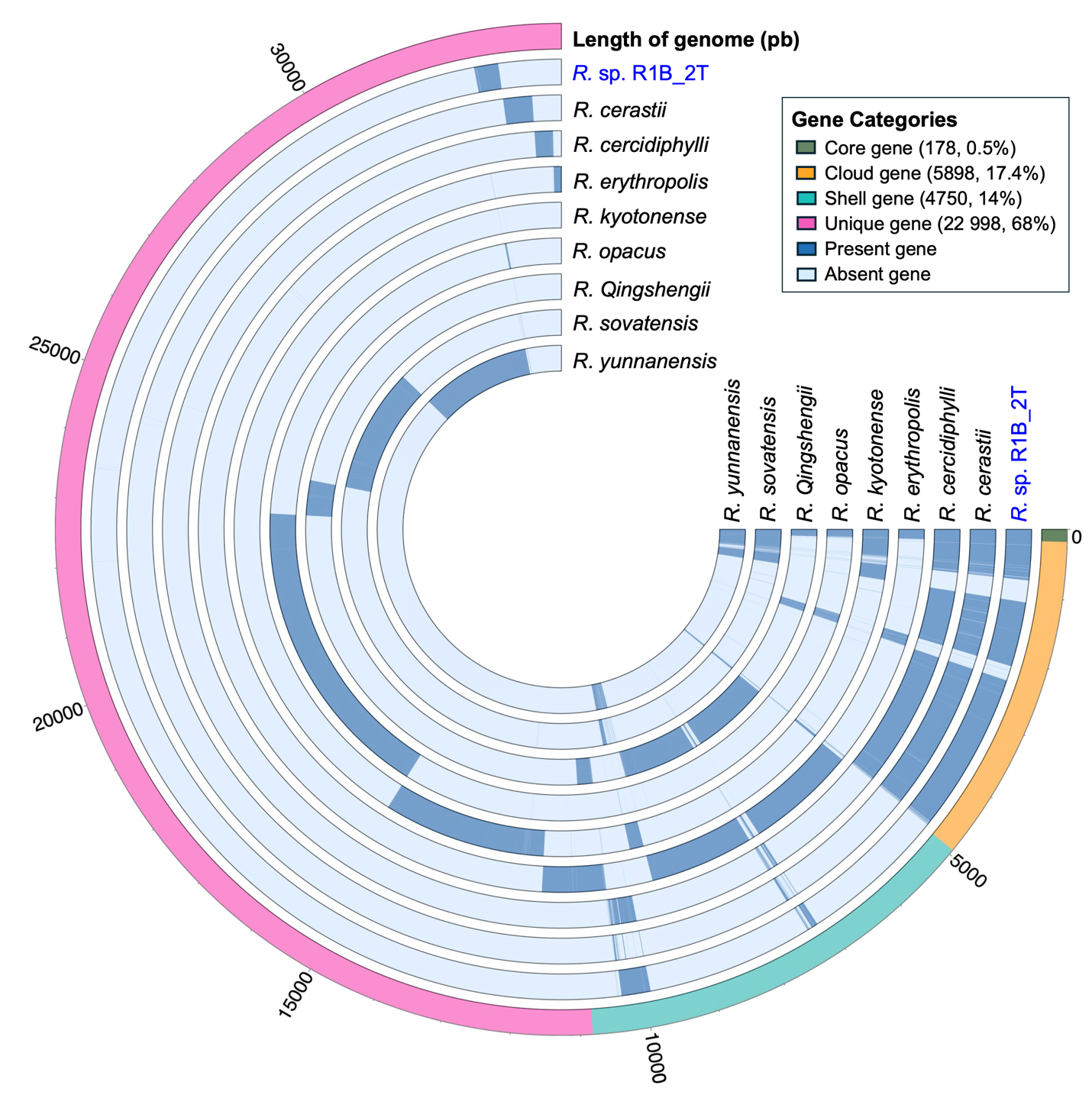
**

**Fig. S1. Pangenome of 10 *Rhodococcus* species**. The circular pangenome compares 9 genomes of referenced species of *Rhodococcus* with *Rhodococcus.* sp. strain R1B_2T. Core gene: genes present in all genomes; cloud gene: genes present in less than 15% of genomes; shell gene: genes present in 15-95% of genomes; and unique gene: genes present in only 1 genome. Genomes information can be found in **Table S3**.

**
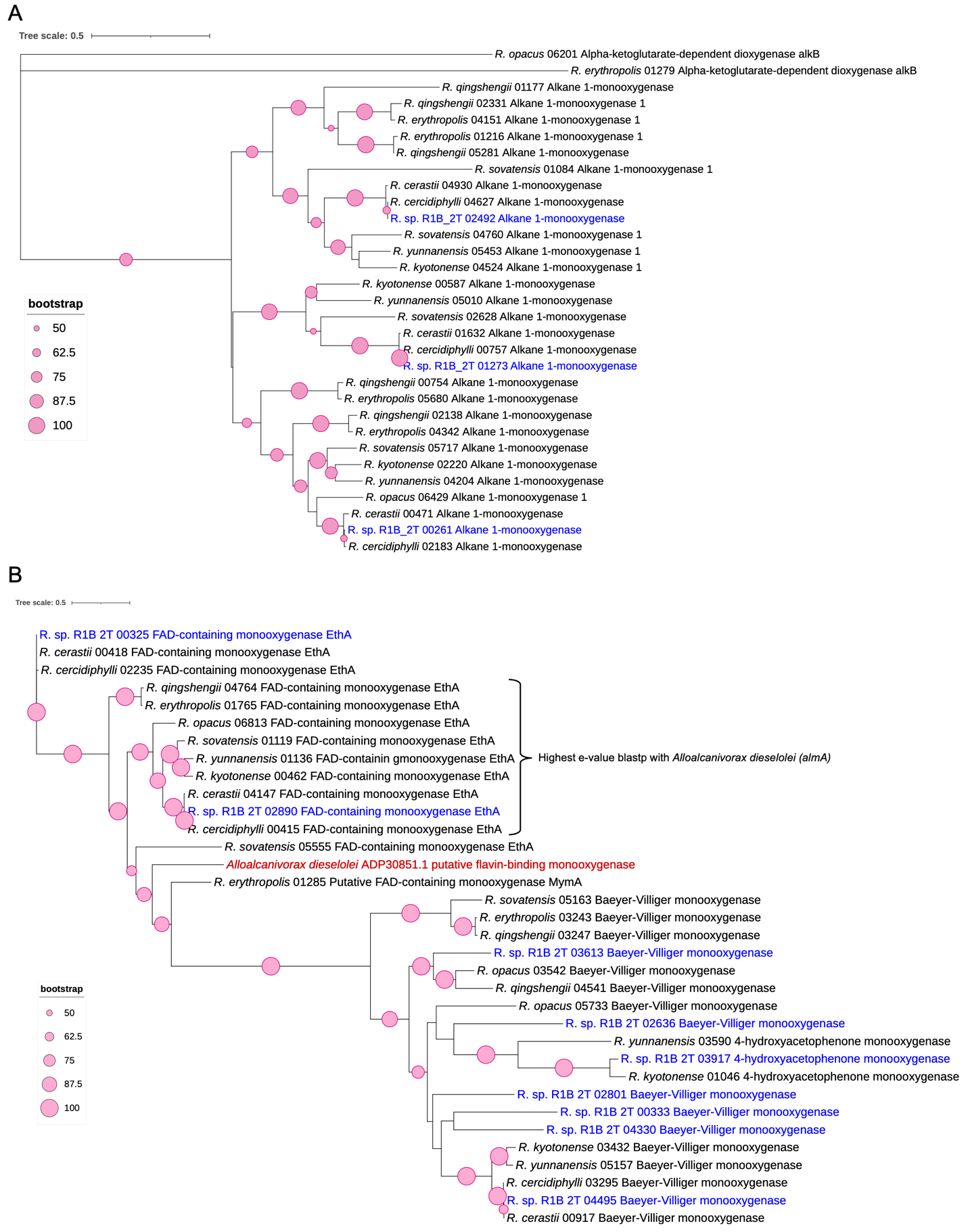
**

**Fig. S2. A Maximum Likelihood *alkB* and *almA* genes trees generated using RAxML**. (**A**) *alkB* genes phylogenetic tree constructed with 31 nucleic acid sequences of 1390 bp aligned along with 8 genomes of different species of *Rhodococcus* and with novel strain R1B_2T. (**B**) *almA* genes phylogenetic tree constructed with 34 amino acid sequences of 560 aa aligned along with 8 genomes of different species of *Rhodococcus* and with novel strain R1B_2T. The trees were constructed with bootstrap support calculated over 1,000 repetitions. Only bootstrap values greater than 50 (out of 100) are displayed. Blastp results for *almA* genes can be found in **Table S4**.


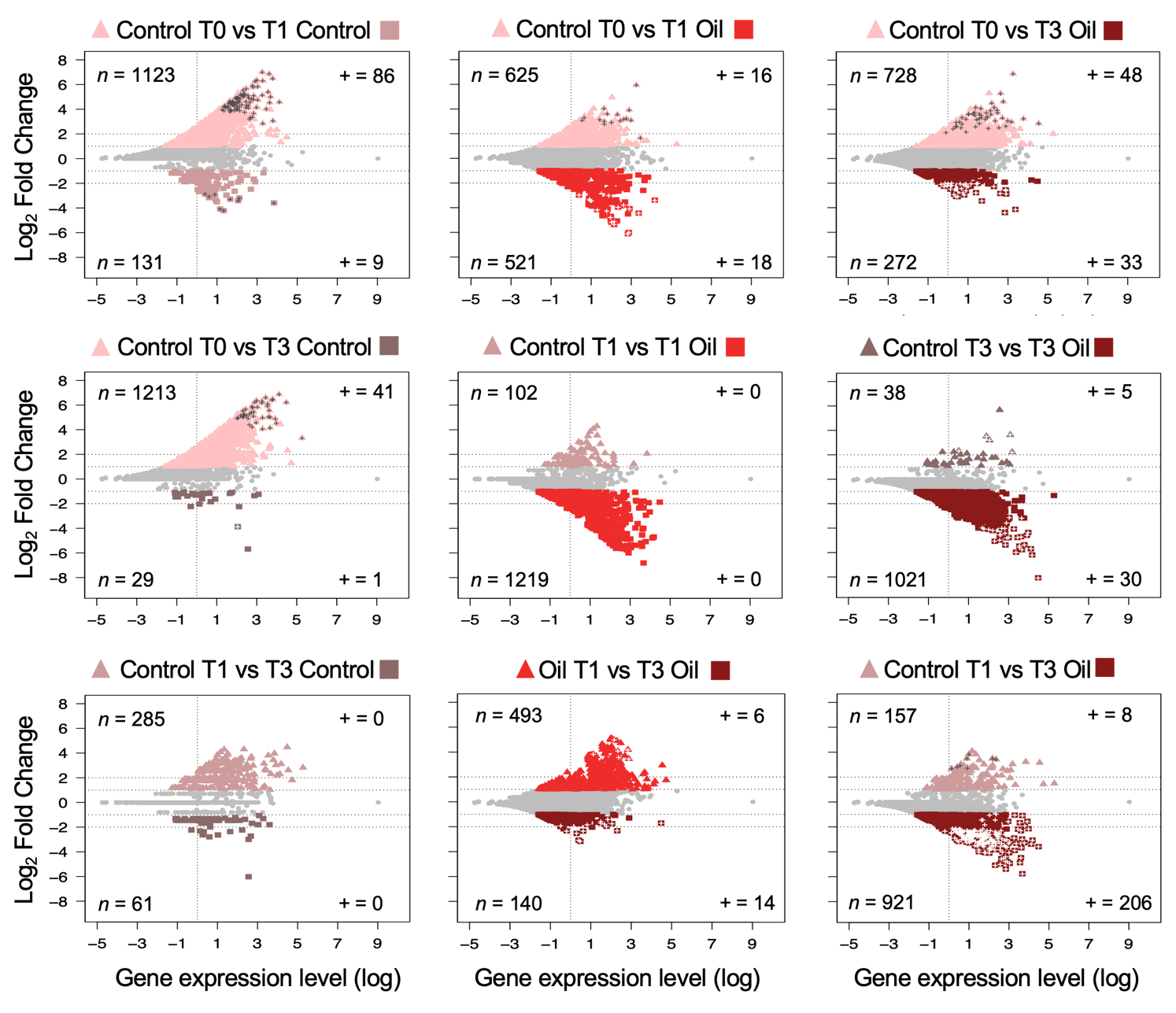


**Fig. S3. Differential expression genes (DEGs) between time of sampling (T0, T1 and T3) with and without oil.** Scatter plots show the comparison between months (T), T1 – 1 month; T3 – 3 months) of cells exposed either with ultra-low sulfur fuel oil (ULSFO) or without oil (control). Each point represents a unigene. Points greater than 1 of log_2_ Fold Change (FC) indicate up-regulated genes and lower than -1 indicate down-regulated genes. Color points indicate differential expressed genes (DEGs). Symbol “+” indicate only filtered DEGs with an Benjamini-Hochberg-adjusted *p*-value <0.05.


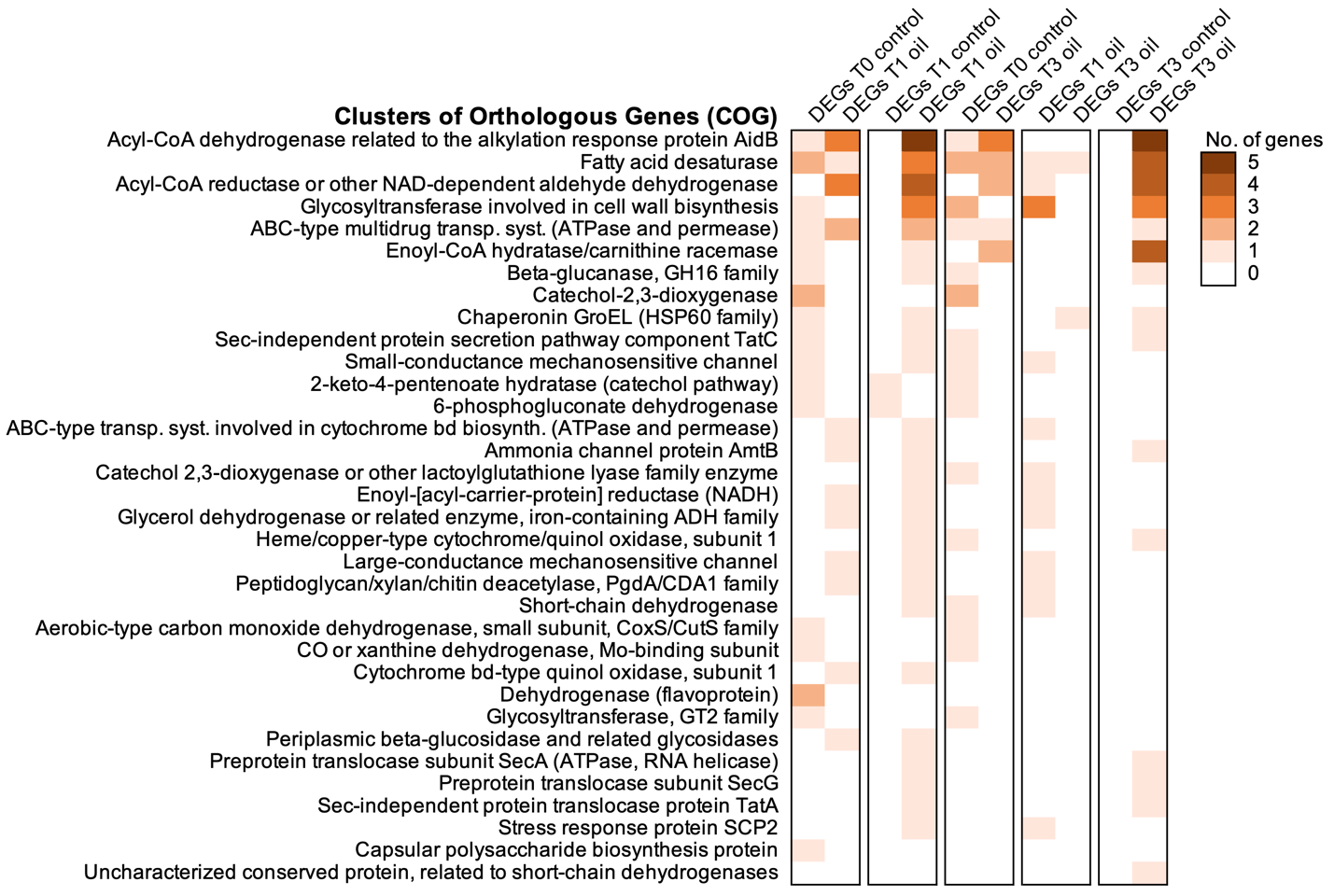


**Fig. S4. Top expression of differentially expressed genes (DEGs) annotated with Clusters of Orthologous Genes (COG) between comparisons of time and with or without the presence of ULSFO.** A complete list of genes and annotations can be found in **Table S8**.

**
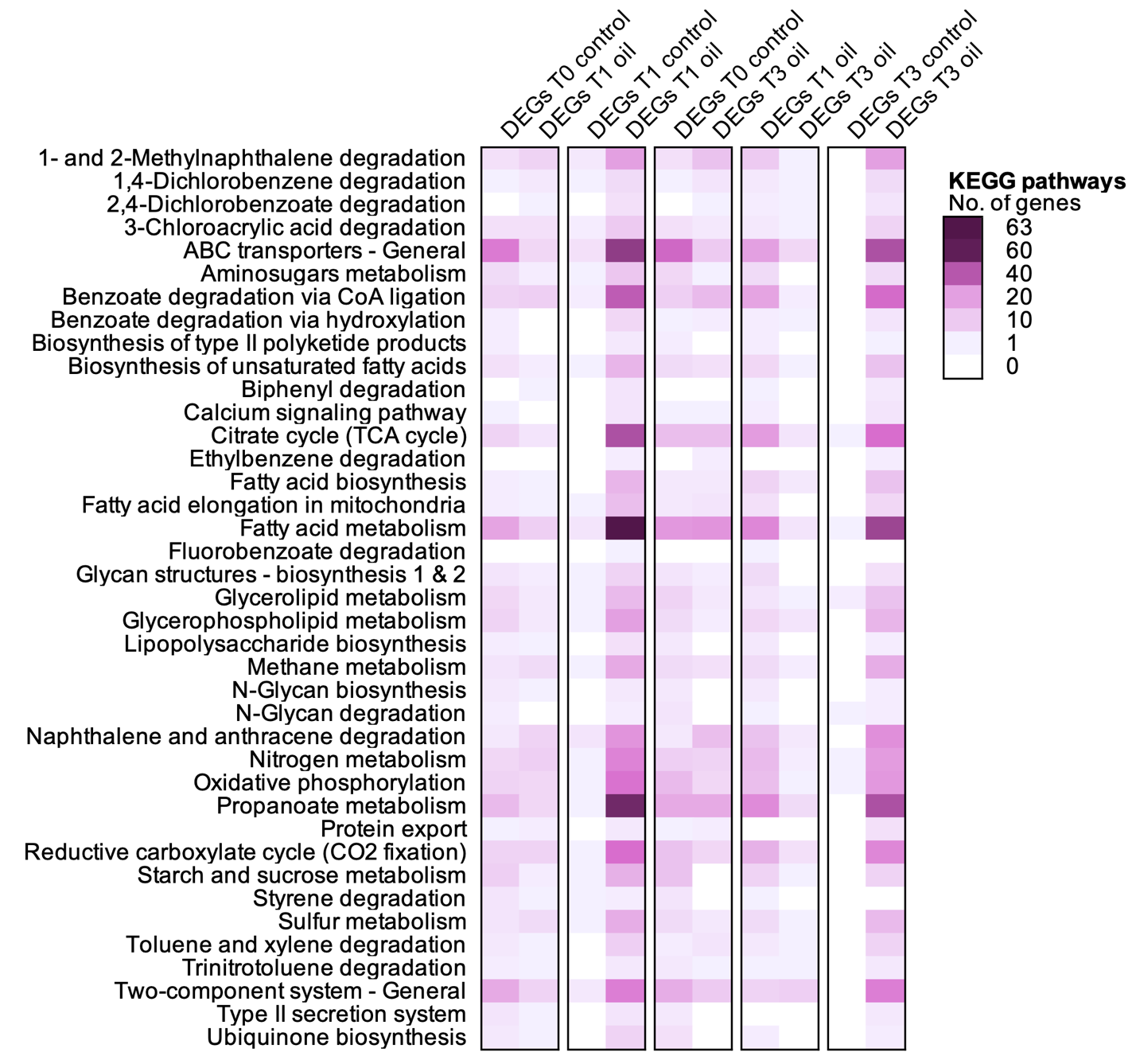
**

**Fig. S5. Top expression of differentially expressed genes (DEGs) annotated with KEGG Orthology (KO) between comparisons of time and with or without the presence of Ultra-Low Sulfur Fuel Oil (ULSFO).** A complete list of genes and annotations can be found in **Table S9**.

**
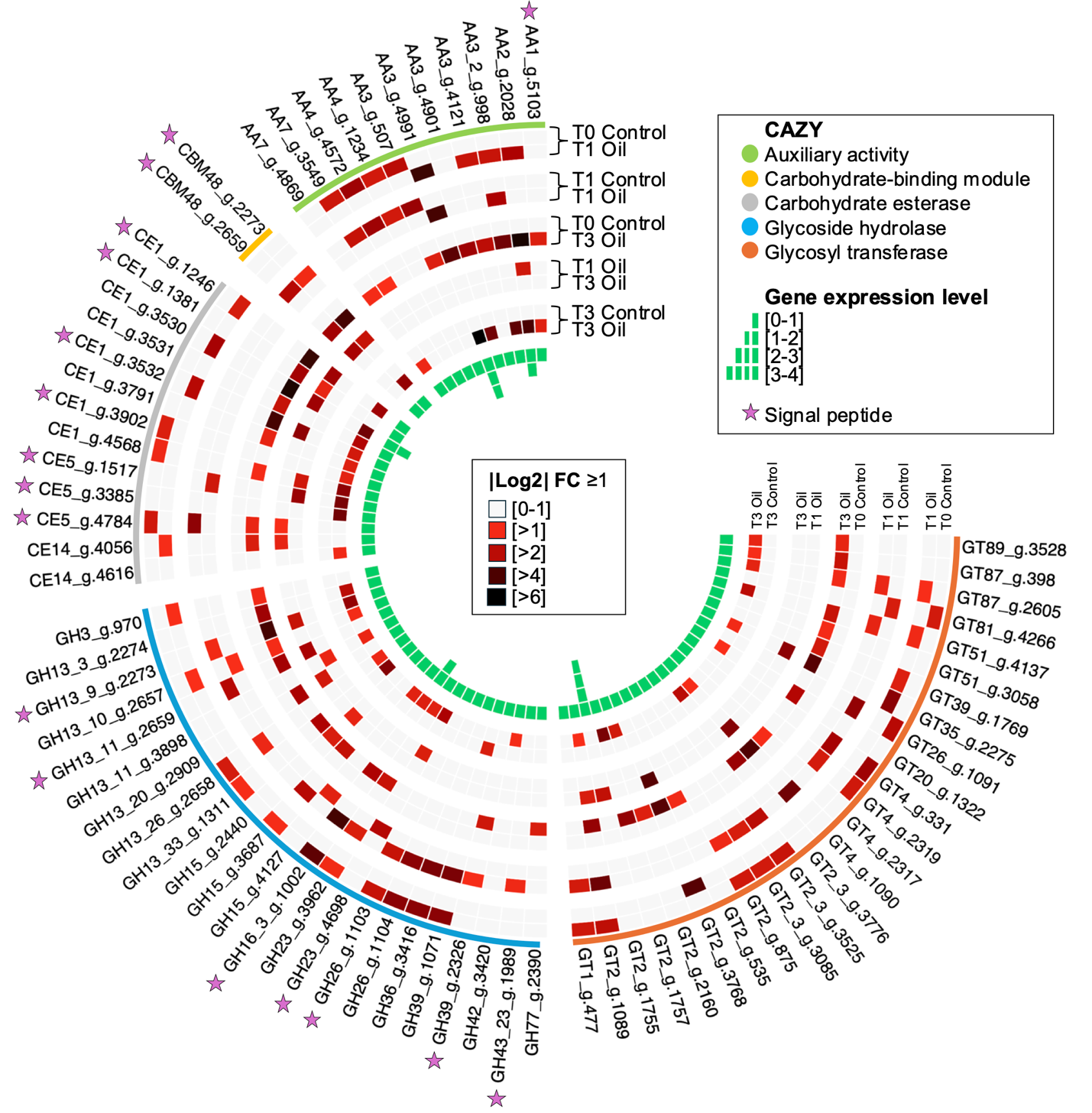
**

**Fig. S6. Circular heatmap of differentially expressed genes (DEGs) annotated as** **carbohydrate-active enzymes (CAZy) categories.** The heatmap depicts expression profiles based on log2 fold changes across sampling timepoints (T0, T1 - 1 month, T3 - 3 months) under conditions with and without Ultra-Low Sulfur Fuel Oil (ULSFO). The outer circle color-codes each DEG according to its specific CAZy category. Expression intensity is represented by color gradient, with darker shades indicating higher expression levels. **Table S12** provides a comprehensive list of all genes with their annotations.

**
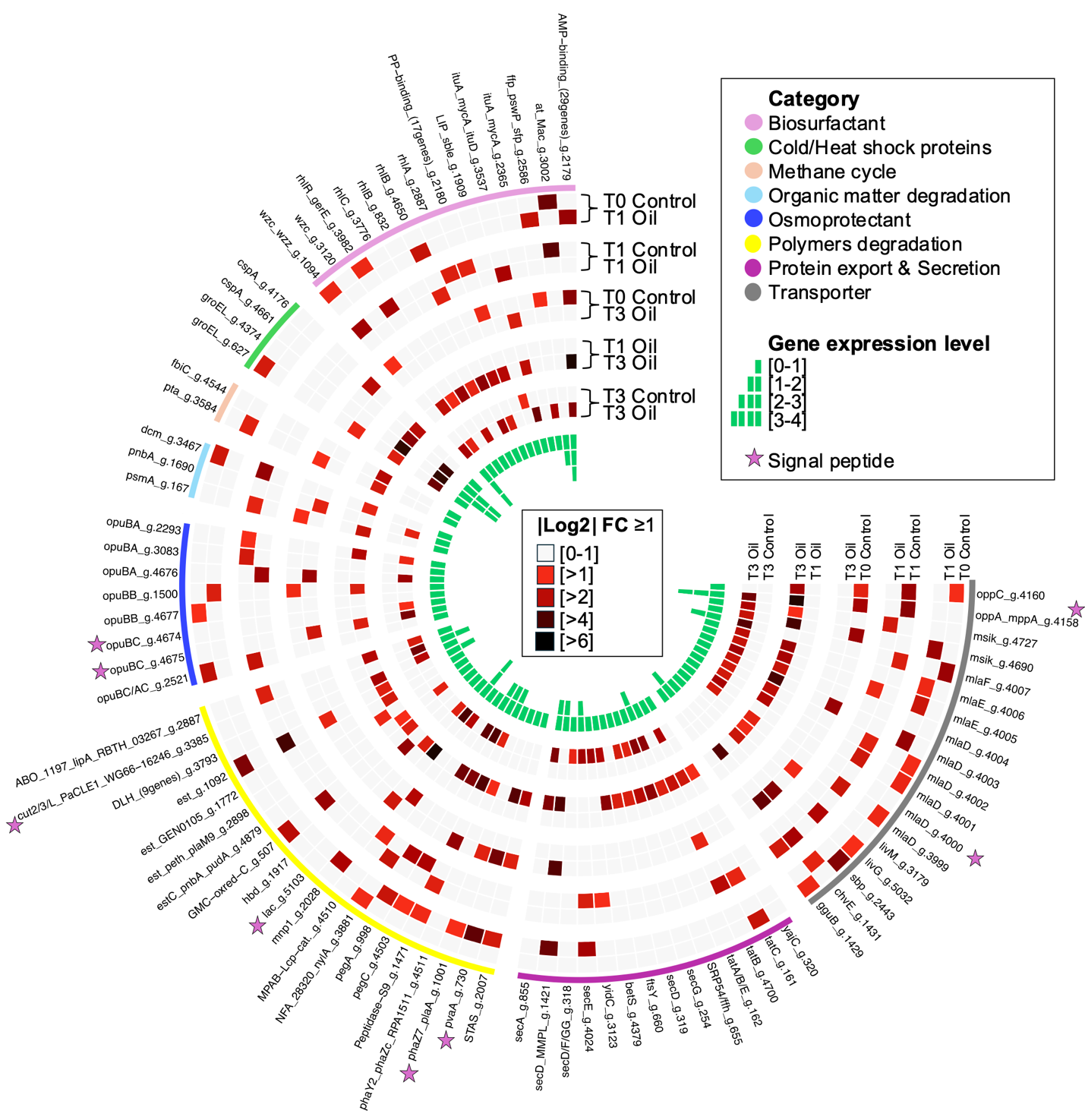
**

**Fig. S7. Circular heatmap of differentially expressed genes (DEGs) annotated as** **different pathway categories.** The heatmap depicts expression profiles based on log2 fold changes across sampling timepoints (T0, T1 - 1 month, T3 - 3 months) under conditions with and without Ultra-Low Sulfur Fuel Oil (ULSFO). The outer circle color-codes each DEG according to its specific pathway annotation category. Expression intensity is represented by color gradient, with darker shades indicating higher expression levels. **Table S13** provides a comprehensive list of all genes with their annotations.
